# Chryseochelins—structural characterization of novel citrate-based siderophores produced by plant protecting *Chryseobacterium* spp.

**DOI:** 10.1093/mtomcs/mfad008

**Published:** 2023-02-15

**Authors:** Karoline Rehm, Vera Vollenweider, Shaohua Gu, Ville-Petri Friman, Rolf Kümmerli, Zhong Wei, Laurent Bigler

**Affiliations:** University of Zurich, Department of Chemistry, Winterthurerstrasse 190, 8057 Zurich, Switzerland; University of Zurich, Department of Quantitative Biomedicine, Winterthurerstrasse 190, 8057 Zurich, Switzerland; Center for Quantitative Biology, Academy for Advanced Interdisciplinary Studies, Peking University, Beijing 100871, China; Peking-Tsinghua Center for Life Sciences, Academy for Advanced Interdisciplinary Studies, Peking University, Beijing 100871, China; University of York, Department of Biology, Wentworth Way, York YO10 5DD, UK; University of Zurich, Department of Quantitative Biomedicine, Winterthurerstrasse 190, 8057 Zurich, Switzerland; Jiangsu Provincial Key Lab for Organic Solid Waste Utilization, Jiangsu Collaborative Innovation Center for Solid Organic Waste Resource Utilization, National Engineering Research Center for Organic-based Fertilizers, Nanjing Agricultural University, Nanjing, PR China; University of Zurich, Department of Chemistry, Winterthurerstrasse 190, 8057 Zurich, Switzerland

**Keywords:** amphiphilic, MS/MS fragmentation, NMR, pathogen control, rhizobacteria, UHPLC–HR–MS

## Abstract

Bacteria secrete siderophores whose function is to acquire iron. In recent years, the siderophores of several *Chryseobacterium* species were shown to promote the health and growth of various plants such as tomato or rice. However, the chemical nature of *Chryseobacterium* siderophores remained unexplored despite great interest. In this work, we present the purification and structure elucidation by nuclear magnetic resonance (NMR) spectroscopy and tandem mass spectrometry (MS/MS) of chryseochelin A, a novel citrate-based siderophore secreted by three *Chryseobacterium* strains involved in plant protection. It contains the unusual building blocks 3-hydroxycadaverine and fumaric acid. Furthermore, the unstable structural isomer chryseochelin B and its stable derivative containing fatty acid chains, named chryseochelin C, were identified by mass spectrometric methods. The latter two incorporate an unusual ester connectivity to the citrate moiety showing similarities to achromobactin from the plant pathogen *Dickeya dadantii*. Finally, we show that chryseochelin A acts in a concentration-dependent manner against the plant-pathogenic *Ralstonia solanacearum* strain by reducing its access to iron. Thus, our study provides valuable knowledge about the siderophores of *Chryseobacterium* strains, which have great potential in various applications.

## Graphical Abstract

**Graphical Abstract:**
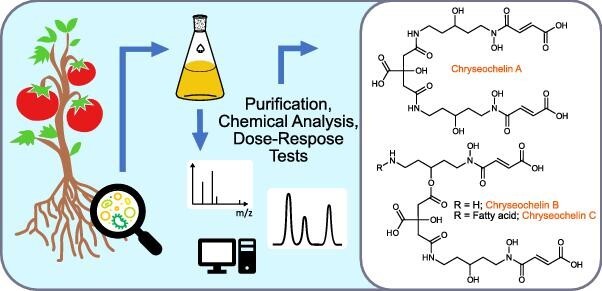
Investigation of novel citrate-based siderophores produced by *Chryseobacterium* spp.: Structures of chryseochelin A, B and C identified by MS/MS fragmentation and proposed structure of chryseochelin A confirmed by NMR.

## Introduction

Microbial organisms such as bacteria or fungi often form mutually beneficial relationships with plants.^1–4^ Plants secrete various nutrients and lysates via their roots that can be utilized by colonizing rhizobacteria. In return, these microorganisms can enhance plant resistance against abiotic stresses like drought or salinity, protect plants from pathogens by secreting secondary metabolites, and improve plant nutrition by, e.g. fixing nitrogen or sequestering insoluble iron.^5–7^

**Fig. 1:**
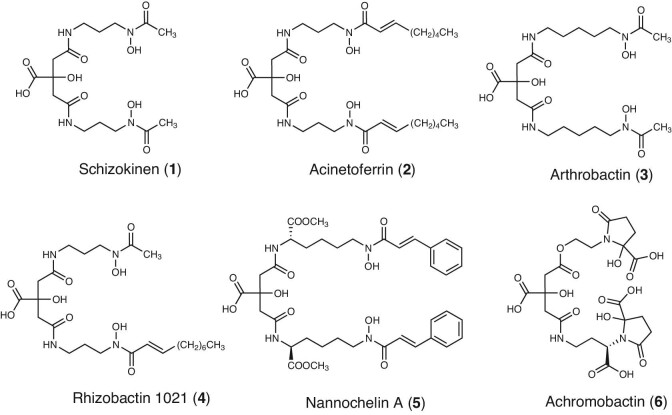
Examples of citrate-based siderophores of various soil bacteria: schizokinen (**1**) from *Bacillus megaterium*,^13^ acinetoferrin (**2**) from *Acinetobacter haemolyticus*,^14^ arthrobactin (**3**) from *Arthrobacter pascens*,^15^ rhizobactin 1021 (**4**) from *Rhizobium meliloti*,^16^ nannochelin A (**5**) from *Nannocystis exedens* strain^17^, and achromobactin (**6**) from *Dickeya dadantii*.^18^

One class of secondary metabolites that has been associated with both improved nutrition and plant protection are sidero-phores, iron-chelating molecules. Although iron is essential for many biological processes, its concentration in the rhizosphere is extremely low due to the insolubility of inorganic iron minerals. Therefore, bacteria secrete siderophores into their environment to dissolve and bind iron, which is then delivered to the cell surface by diffusion and is internalized via cognate receptors.^8^,^9^ In order to gain a competitive advantage, most bacterial strains produce a specific molecule that can only be taken up by individuals that carry the specific receptor, which are typically closely related strains or clonemates. Due to this competitive pressure, several hundred different siderophore structures have been discovered in the past 70 yr, making siderophores a chemically diverse class of molecules.^10^ Siderophores can be classified based on the iron-chelating ligands that are present in their chemical structure such as catecholate, hydroxamate and/or α-hydroxy carboxylate units.^10^,^11^ For instance, several soil bacteria produce citrate-derived siderophores (Fig. 1), which contain a citrate moiety as well as two hydroxamate units. Those are commonly connected by either two 1,3-diaminopropane, 1,5-diaminopentane or lysine groups. In a few cases, unusual variants exist such as the incorporation of 2-oxoglutaric acid groups instead of hydroxamate units.^12^

Plants often benefit from the secreted bacterial siderophores in multiple ways. Firstly, siderophores can increase the bioavailability of iron and thereby enhance plant growth. Additionally, siderophores can lock iron away from plant pathogens that lack the matching receptors for uptake.^19^ Both of these plant beneficial effects have been demonstrated to take place with *Chryseobacterium* spp. The genus *Chryseobacterium* belongs to the *Flavobacteriaceae* family and is commonly found in soil, water, and clinical sources.^20^,^21^ In the study of Radzik *et al.*,^22^ an increased plant yield was obtained by supplementing tomato plants with siderophore-rich supernatant from *Chryseobacterium* C138 that promoted iron uptake of the plants via their roots. Similar results were found for sunflower and rice plants that were inoculated with siderophore-producing *Chryseobacterium* strains.^23–25^ The antagonistic effect of *Chryseobacterium*’s siderophores has been demonstrated against the plant-pathogenic *Ralstonia solanacearum*, where they effectively protected tomato plants from infection.^26^,^27^

Despite the potential benefits of *Chryseobacterium* siderophores as biofertilizers and pathogen control agents, the chemical nature of these iron chelators has remained unclear due to difficulties in identifying and elucidating its structure. Therefore, studies on the exact mechanism of action, direct detection and quantification of siderophore effects, and chemical synthesis for commercial applications have not yet been conducted.

In this work, we investigated the siderophores secreted by three *Chryseobacterium* strains whose genus-level taxonomic identification and ability to protect tomato plants from *R. solanacearum* infections have been previously reported.^26^ For this purpose, an untargeted ultra-high performance liquid chromatography mass spectrometry (UHPLC-MS) method was developed in order to screen siderophores in bacterial supernatants. MS/MS fragmentation and NMR were then applied for their structure elucidation. To confirm the growth-suppressing activity of *Chryseobacterium* siderophores, we tested a purified siderophore against the plant-pathogenic strain *R. solanacearum* under iron limitation in a dose–response experiment. Our results were then discussed with regard to siderophores produced by other bacteria such as *Dickeya dadantii*. This was done with the aim of providing a biological overview and highlighting potential research and application areas for the siderophores produced by *Chryseobacterium* spp.

## Materials and methods

### Chemicals and reagents

Acetonitrile (CH_3_CN), methanol (MeOH) and isopropanol were obtained from Biosolve (ULC grade, Valkenswaard, Netherlands) and formic acid from Fluka (LC-MS grade, Buchs, SG, Switzerland). Ultrapure water (<2 ppb TOC) was produced using a Milli-Q Advantage A10 water purification system (Merck, Bedford, MA, USA). Iron(III) chloride hexahydrate of 99+% purity was bought from ACROS Organics (Fair Lawn, NJ, USA). For mass and ion mobility calibration, a 1:1 mixture of Agilent ESI-L low concentration tune mix (Santa Clara, CA, USA) and a 10 mM sodium formate solution was prepared. The 10 mM sodium solution contained 1 M NaOH (250 μL) and formic acid (50 μL) in 50% isopropanol (25 mL). A total of 99.8% D_2_O for NMR measurements was obtained from Sigma Aldrich (Buchs). Tryptic soy broth (TSB), lysogeny broth (LB), and casamino acid solution (CAA) were used as growth media for bacterial assays. CAA (1 L) was made from vitamin free casein acid hydrolysate (10.0 g), K_2_HPO_4_ · 3H_2_O (1.18 g) and MgSO_4_ · 7H_2_O (0.25 g). The listed ingredients, TSB and LB were bought from Sigma Aldrich (Buchs).

### Bacterial strains

The three *Chryseobacterium* isolates, CHR2, CHR5, and CHR6, are representative members of a collection of 120 *Chryseobacterium* spp. sampled from the soil of tomato fields in China. The detailed description of the sampling, isolation, and characterization can be found in the work of Gu *et al.*^26^ Using the chrome azurol S assay, it was demonstrated that the bacterial strains produced siderophores and that their siderophore-containing supernatants were capable of inhibiting the plant pathogen *R. solanacearum* under iron-limited conditions.^26^ Genus-level taxonomic identification was obtained by sequencing the 16S rRNA gene using the universal primers F27 (5′-AGAGTTTGATCATGGCTCAG-3′) and R1492 (5′-TACGGTTACCTTGTTACGACTT-3′).

The plant-pathogenic strain *R. solanacearum* K60 was used for the dose–response experiments.^28^ Experiments were carried out in the laboratory of Prof. Leo Eberl (Department of Plant and Microbial Biology, University of Zurich) who has the necessary authorization to work with this pathogen.

### *Chryseobacterium* spp. supernatant generation

Liquid cultures of the three *Chryseobacterium* strains CHR2, CHR5, and CHR6 were grown according to an adapted protocol described in the paper of Gu *et al.*^26^ Briefly, 5 μL of gycerol stocks (kept at −80°C) were inoculated into 195 μL TSB in a 96-well plate and incubated at 28°C for 22 h (170 rpm). A total of 10 μL of the overnight culture were then transferred into a new 96-well plate containing 190 μL CAA and incubated for 48 h at 28°C (170 rpm). The liquid cultures were then centrifuged at 10 733 rcf for 10 min at room temperature, and the supernatant was filtered through a 0.22 μm filter before being stored at −20°C. For strain CHR2, the earlier protocol was scaled up to achieve a final supernatant volume of 450 mL (2 overnight plates and 28 CAA plates in total). This supernatant was then used for the siderophore purification.

### Sample preparation for siderophore screening

For the siderophore screening, three biological replicates of bacterial supernatant were prepared for each bacterium and measured by UHPLC–MS. A total of 100 μL filtered supernatant was frozen by liquid nitrogen and lyophilized in a vacuum concentrator over several hours (23°C, 3000 rpm) to quench remaining enzymatic activities.^29^ The residue was dissolved in 150 μL 40% aq. MeOH and transferred to a vial. These samples contained mainly the apo-siderophores, meaning the siderophores not bound to iron. An aliquot of 60 μL was then removed and put into a second vial. This second set of samples was spiked each with 3 μL of a freshly prepared aqueous FeCl_3_ solution (100 mM) in order to obtain the iron complexes of the siderophores chryseochelin A, chryseochelin B, and chryseochelin C.

### UHPLC conditions

Liquid chromatography was performed using a Vanquish Horizon UHPLC system by Thermo Fisher (Waltham, MA, USA) build from a binary pump H, a split sampler HT, and a temperature-controlled column compartment. Chromatographic separation was achieved on an ACQUITY UPLC HSS T3 Column (100 Å, 1.8 μm, 2.1 ×100 mm, Waters, Milford, USA) at 40°C with a flowrate of 0.5 mL/min using H_2_O + 0.1% HCOOH as eluent A and CH_3_CN + 0.1% HCOOH as eluent B. The following gradient was applied: (i) 4% B isocratic from 0.0–0.5 min; (ii) linear increase to 15% B until 4 min; (iii) linear increase to 80% until 7.0 min; (iv) linear increase to 100% until 7.1 min; (v) holding 100% B until 10.0 min; (vi) back to the starting conditions of 4% B until 10.1 min; and (vii) equilibration for 4.5 min until the next run.

### MS instrumentation

A timsTOF Pro hybrid quadrupole-time-of-flight mass spectrometer employing trapped ion mobility spectrometry (TIMS) produced by Bruker (Bremen, Germany) recorded MS and MS/MS data in positive electrospray ionization (ESI) mode. Source parameters were set as followed: End plate offset of 500 V, capillary voltage of 4500 V, nebulizer pressure of 2.2 bar (N_2_), and heated dry gas flow of 10.0 L/min with a temperature of 220°C. Mass and collision cross section calibrations were conducted with an Agilent low concentration tune mix diluted in a sodium formate solution that was injected using a 6-port valve with a 20 μL loop prior to each sample measurement. The relevant TIMS parameters are stated according to the recommendations for reporting ion mobility measurement settings^30^: A ramp time of 100 ms with an inverse reduced mobility range of 0.55–1.90 1/*K*_0_, a target count of 5 million for ion charge control (ICC), radiofrequency of the TIMS ion funnels of 250 Vpp. The average TIMS cartridge tunnel-in pressure and the tunnel-out pressure were 2.6 and 0.8 mbar, respectively. As a drift gas, nitrogen of at least 4.5 purity was used that was additionally purified by a HC Big Supelpure HC Hydrocarbon Trap from Sigma Aldrich (Buchs). MS/MS spectra were acquired by parallel accumulation–serial fragmentation with a collision energy of 25 or 35 eV. DataAnalysis 5.2 software (Bruker) was used for data evaluation. A mass error up to 5 ppm was tolerated.

### Chryseochelin A purification

A total of 450 mL bacterial supernatant was prepared under iron limited conditions as described in the previous section. In a first step, the supernatant was frozen by liquid nitrogen and then lyophilized overnight. The dried residue was reconstituted in 80 mL MeOH and the solution was filtered over a filter paper. Afterward, the organic solvent was evaporated under a gentle stream of nitrogen. The dried extract was then dissolved in 5 mL ultrapure H_2_O. This solution containing the siderophore chryseochelin A was centrifuged for 10 min (4°C, 14 000 rpm) and transferred into a new Eppendorf tube. The crude siderophore was then purified twice by reversed-phase high pressure liquid chromatography (RP–HPLC) on a preparative Triat C18 column (150 × 30 mm, 5 μm, YMC-Actus) and then on an analytical CORTECS C18 column (150 × 4.6 mm, 2.7 μm, Waters) while monitoring the absorption wavelength at 270 nm. Detailed chromatographic conditions can be found in the supplementary (Supplementary Figs. S1 and S2). The final pure siderophore (3.1 mg), chryseochelin A, was stored as a white powder at −20°C.

### Structure elucidation and chemical characterization

UHPLC–HR–MS was applied to determine accurate masses of siderophores and to predict their molecular formula. MS/MS fragmentation spectra were collected with the conditions described earlier. NMR data of purified chryseochelin A were measured in D_2_O on a Bruker AV-600 MHz instrument equipped with a TCI CryoProbe. Thereby, the following experiments were included: ^1^H, ^13^C, distortionless enhancement by polarization transfer (DEPT) 90 and 135, heteronuclear single quantum correlation spectroscopy (HSQC), correlation spectroscopy (COSY), heteronuclear multiple bond correlation (HMBC), total correlation spectroscopy (TOCSY), and nuclear overhauser and exchange spectroscopy (NOESY). Ultraviolet-visible (UV/VIS) spectra of chryseochelin A, its ferric complex and photoproduct were recorded on a NanoDrop 2000c spectrophotometer (Thermo Scientific).

### Photolysis

The Fe(III)–chryseochelin A complex (0.2 mM) was prepared in 20 mM phosphate buffered H_2_O (pH 7.0) and exposed to sunlight for 3 h at 25°C. The photolysis reaction was assessed by UV/VIS spectroscopy and by ESI–MS.

### Dose–response curves

*Ralstonia solanacearum* K60 overnight cultures were grown in 4 mL LB at 30°C and 220 rpm agitation. Cultures were washed twice with 0.8% NaCl and adjusted to an optical density at 600 nm (OD_600_) of 0.1. For the dose–response assays, a 25 mg/mL stock solution of the purified chryseochelin A extract was prepared in ultrapure water, which was further diluted to the highest working concentration of 100 μg/mL in either CAA (low iron condition) or CAA supplemented with 50 μM FeCl_3_ (high iron condition). The working stocks were filter sterilized and serially diluted (2-fold dilutions) in nine steps. A total of 198 μL of each dilution was then transferred to a 96-well plate in duplicates and 2 μL of adjusted *R. solanacearum* culture was added. The pure CAA medium (200 μL) without chryseochelin served as a control. The plate was incubated at 30°C statically in a plate reader (MWG Sirius HT, MWG Biotech, Ebersberg, Germany). The plate was shaken for 1 min each time before the OD_600_ was measured (every 20 min for 71 h). The OD_600_ data were then blank corrected and the integral (area under the growth curve) was calculated using the R package Growthcurver.^31^ Integral values were expressed relative to the respective untreated control (0 μg/mL chryseochelin A in either low or high iron condition). For the low iron treatment, a dose–response curve was fitted with a four-parameter logistic regression using the nplr package.^32^ No dose–response curve could be fitted to the growth data from the high iron treatment.

## Result and discussion

### Siderophore screening

**Fig. 2:**
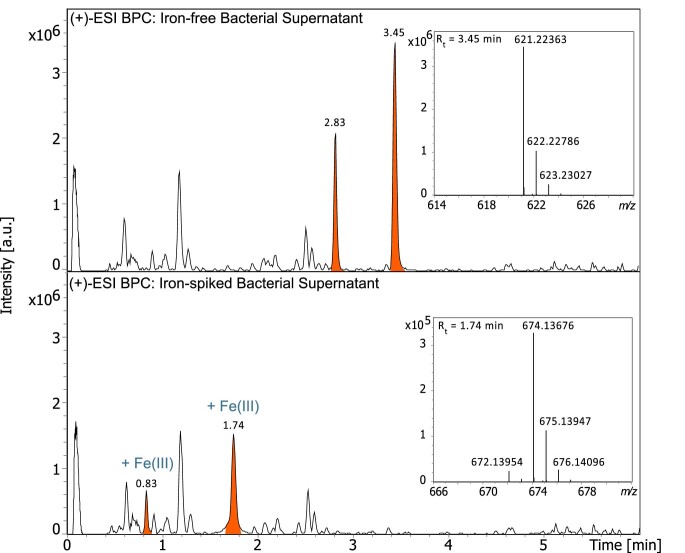
Base peak chromatogram (BPC) (*m*/*z* 50–2000) of *Chryseobacterium* supernatant grown under iron-limited conditions (top) and after spiking it with iron (bottom) together with inserts of the *m*/*z* value of the protonated chryseochelin A (*R_t_* = 3.35 min) and its iron complex (*R_t_* = 1.74 min).

UHPLC–MS analysis revealed that all three *Chryseobacterium* strains produced identical siderophores under iron-limited conditions. Figure 2 shows the chromatographic separation of the supernatant of *Chryseobacterium* before and after spiking it with an aqueous Fe(III) solution. In iron-limited supernatant, the chromatogram is dominated by two peaks, chryseochelin A at 3.45 min and chryseochelin B at 2.83 min, both corresponding to a *m*/*z* value of 621 ([M + H]^+^). After addition of iron, these peaks shifted (1.74 min for chryseochelin A and 0.83 min for chryseochelin B) and the ionization efficiency of both compounds decreased noticeably. A new *m*/*z* value of 674 was obtained matching the [M − 2H + Fe(III)]^+^ adduct. The presence of the iron atom was confirmed by its characteristic isotopic distribution.

In this way, chryseochelin A and B were identified as the main siderophores produced by the *Chryseobacterium* strains. Their two molecular formulae were predicted as C_24_H_36_N_4_O_15_, taking into account the seven golden rules of molecular formula prediction.^33^ No reasonable match was found in our in-house siderophore library containing over 450 known siderophores or in general databases such as the Natural Products Atlas or PubChem. Hence, we concluded that the detected chryseochelin A and chryseochelin B were novel compounds that had not yet been reported in the literature. We chose the name chryseochelin for this new suite of siderophores derived from the bacterial genus (*Chryseobacterium*) and their metal-chelating function. Although “-bactin” is another popular suffix for molecules of bacterial origin, we decided against it due to potential confusion with an unrelated siderophore called “chrysobactin” produced by *Dickeya chrysanthemi*.^34^

### Structural elucidation of chryseochelin A

**Fig. 3:**
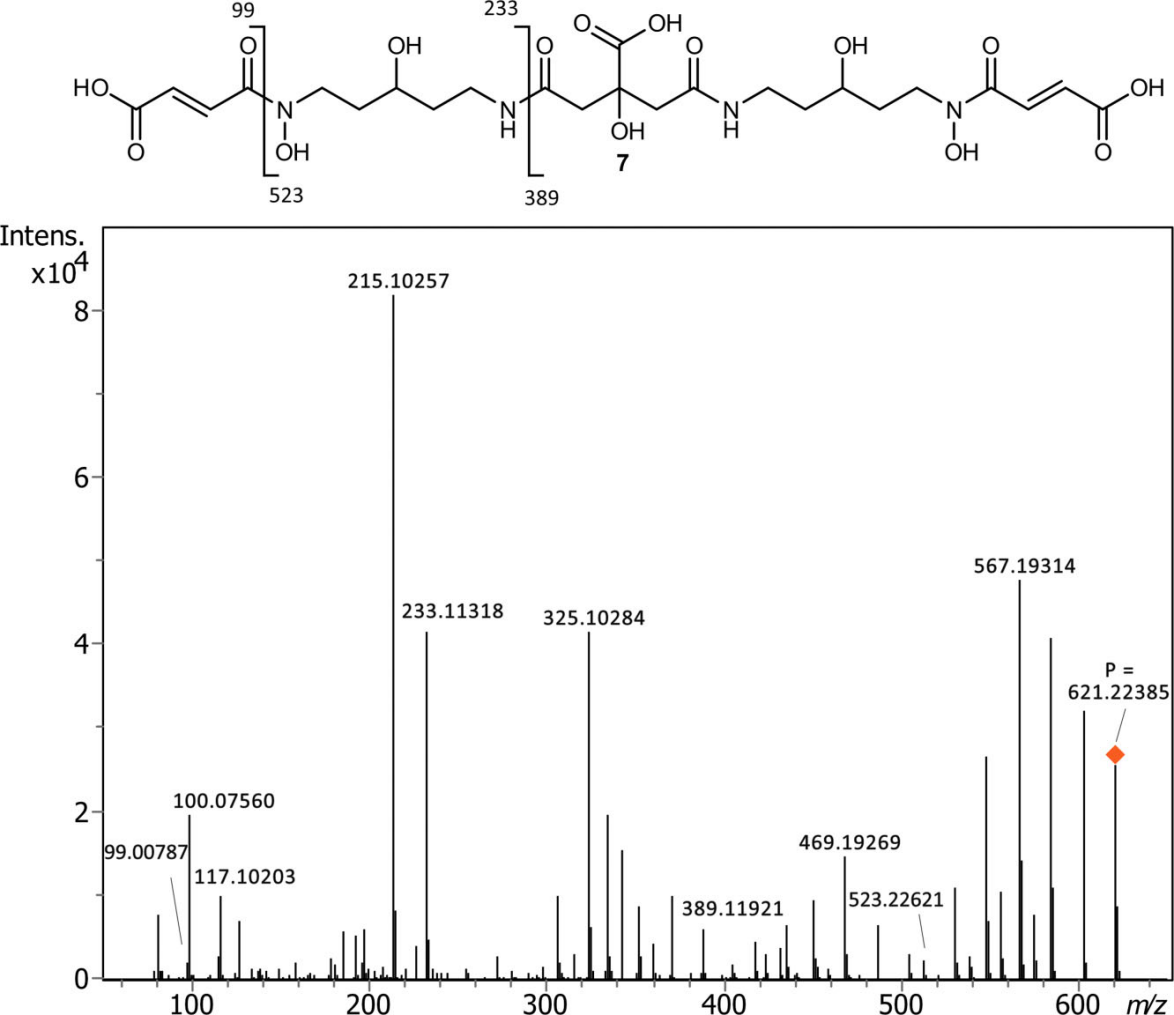
MS/MS spectrum of chryseochelin A (**7**) (precursor ion *m*/*z* 621) at a collision energy of 35 eV. A peak list is available in the supplementary (Supplementary Table S1).

We successfully isolated a pure fraction of the main siderophore, chryseochelin A (**7**), from the bacterial supernatant of our *Chryseobacterium* strains (see Supplementary Fig. S1 and S2). This compound is a citrate-based di-hydroxamate siderophore with dual fumaric acid appendages (Fig. 3). Its structural elucidation was conducted by HR–MS/MS fragmentation and NMR, which will be discussed in more detail hereafter.

UHPLC–HR–MS revealed an exact mass of *m*/*z* 621.22385 for the [M + H]^+^ ion agreeing with the proposed molecular formula C_24_H_36_N_4_O_15_ (1.8 ppm). Such a high carbon-to-oxygen ratio is typically found only for citrate-based siderophores.^11^ A characteristic fragmentation pattern was obtained by MS/MS fragmentation at a collision energy of 35 eV, depicted in Fig. 3. Multiple H_2_O (−*m*/*z* 18) and CO_2_H_2_ (−*m*/*z* 46) losses from the parent ion and other major fragments indicate the presence of hydroxy groups and a citrate moiety, as also observed by Gauglitz *et al.* for the structurally similar ochrobactins.^35^ By breaking the amide bond at the citrate moiety, the main fragment pair *m*/*z* 233.11318 (C_9_H_17_N_2_O_5_^+^, 0.1 ppm) and 389.11921 (C_15_H_21_N_2_O_10_^+^, −0.4 ppm) are obtained revealing the connectivity of the molecular substructures. A fumaric acid fragment appears at *m*/*z* 99.00787 (C_4_H_3_O_3_^+^, −2.0 ppm) with a weak signal for its counter fragment at *m*/*z* 523.22621 (C_20_H_35_N_4_O_12_^+^, −3.1 ppm). The 3-hydroxycadaverine moiety yields the characteristic fragments at *m*/*z* 100.07560 (C_5_H_10_NO^+^, 0.9 ppm) and 117.10203 (C_5_H_13_N_2_O^+^, 1.8 ppm).

**Fig. 4:**
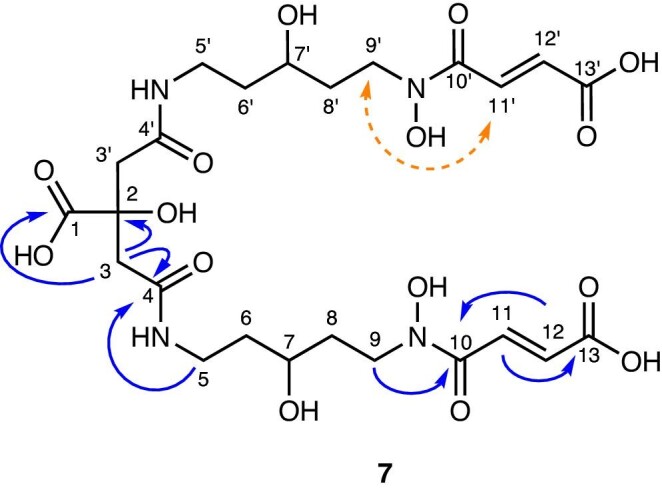
Structure of chryseochelin A (**7**) with indication of selected HMBC (plain arrow, H/C) and NOESY (dashed arrow) correlations essential for the structure elucidation.

The exact structure elucidation was carried out by NMR. The ^1^H and ^13^C NMR spectra of chryseochelin A were measured in D_2_O (≥99.8% atom % D). Chemical shifts are listed in Table 1 and the corresponding assigned positions are visualized in Fig. 4. All 1D and 2D spectra are available in the supplementary (Supplementary Figs. S3–S9). A high similarity of the citrate framework to other citrate-based siderophores such as synechobactins, orchobactin, or acinetobactin was observed.^14^,^35^,^36^ The presence of a citrate moiety was visible in ^1^H by the characteristic doublets at δ_H_ 2.73 and 2.58 ppm and a coupling constant of 14.7 Hz, which belong to the chemically unequal protons at position C3/C3′ due to the stereogenic center at C2. In the range of 1.5–3.8 ppm, several multipletts are seen for CH_2_ groups with unequal protons belonging to the positions C5/C5′, C6/C6′, C8/C8′, and C9/C9′. This complexity results from the chiral center at C7/C7′ to which a hydroxy group is attached. The presence of the hydroxy group is confirmed by a typical ^1^H and ^13^C shift of δ_H_ 3.64 and δ_C_ 66.5 ppm, respectively. Inspecting the signals for C7/C7′ more closely, two very close carbon atom signals at 66.50 and 66.53 ppm with similar intensity are observed and a broad multiplett is obtained for the corresponding proton. Hence, two different chiralities of the hydroxy groups in relation to the citrate center are assumed for both sides of the molecule. Above 6 ppm, the signals of the two protons at the double bond at position C11/C11′ and C12/C12′ arise. The high chemical shift stems from the conjugated carboxylic and hydroxamic acid system as already reported for the identical chemical substructure.^37^,^38^ The splitting of the proton signals at position C9/C9′, C10/C10′, and C11/C11′ can be explained by the isomerism of the hydroxamic acid (see Supplementary Fig. S10) resulting in different chemical shifts.^39^,^40^ This is also confirmed by the appearance of inversed-phased cross peaks in the NOESY spectrum for the exchangeable protons. ^13^C signals were assigned to the corresponding ^1^H atoms via HSQC, and DEPT 90 and 135 measurements confirmed the number of attached protons to a carbon. Carbonyls (C1, C4/C4′, C10, C10′, and C13, C13′) were easily assigned by their high chemical shift (δ_C_ > 160 ppm). ^1^H–^1^H COSY was used to assign the connectivity of the carbon chains (C5/C5′–C9/C9′) confirming the hydroxylated position at carbon C7/C7′. The isolated CH_2_ group at position C3/C3′ and the protons at the double bond (position C10/C10′ and C11/C11′) were shown by TOCSY to be separate spin systems. The spin systems were then connected by ^1^H–^13^C HMBC, revealing a correlation of the protons at position C9/C9′ with carbon C10/C10′ and of the protons at position C5/C5′ and C3/C3′ with carbon C4/C4′. Finally, NOESY was measured to prove the correlation of the protons at position C9/C9′ and C11/C11′, supporting the previous position assignment of the double bond protons based on their chemical shifts.

**Table 1:** ^1^H and ^13^C data of chryseochelin A (**7**)

Position	δ_H_ (ppm)	Multiplicity	δ_C_ (ppm)	
C1			176.84	C=O
C2			74.18	C
C3, C3′	2.73	*d, J* = 14.7 Hz	44.44	CH_2_
	2.58	*d, J* = 14.7 Hz		
C4, C4′			171.31	C=O
C5, C5′	3.22	*m*	36.00	CH_2_
C6, C6′	1.65	*m*	35.41	CH_2_
	1.57	*m*		
C7, C7′	3.64	*m*	66.53	CH
			66.50	
C8, C8′	1.82	*m*	33.00	CH_2_
	1.70	*m*		
C9, C9′^a^	3.82	*m*	45.58	CH_2_
	3.70	*m*		
	3.88	*m*	48.35	
	3.77	*m*		
C10, C10′^a^			165.74	C=O
			162.99	
C11, C11′^a^	7.49	*d, J* = 15.8 Hz	132.06	CH
	7.29	*d, J* = 15.5 Hz	132.06	
C12, C12′	6.67	*d, J* = 15.7 Hz	132.36	CH
C13, C13′			169.57	C=O

^a^Multiple signals due to isomerism of the hydroxamate acid (see Supplementary Fig. S10).

To complete the data set, UV/VIS spectra of chryseochelin A and Fe(III)-chryseochelin A were collected (see Supplementary Fig. S11). The colorless chryseochelin A has a distinct absorption shoulder at 260 nm. Upon addition of iron, an immediate color change to brown is observable indicating the formation of the Fe(III)-complex. A characteristic charge-transfer transition from the hydroxamate to the Fe(III) band is visible at 410 nm as well as an absorption shoulder at 270 nm. Similar absorption wavelengths were observed for acinetoferrin^14^ or rhizobactin 1021^16^ belonging to the same class of citrate-based siderophores.

In this way, the chemical structure of chryseochelin A was reliably elucidated using MS/MS fragmentation spectra, a complete set of NMR data and UV/VIS spectra supported by various references.

### Photolysis of ferric chryseochelin A

Citrate-based siderophores are known to react with sunlight, resulting in the loss of the central carboxylic acid and the formation of a new photoproduct that is still capable of binding iron. In this process, Fe(III) is reduced to Fe(II) but is readily re-oxidized to Fe(III).^35^,^41^

**Fig. 5:**
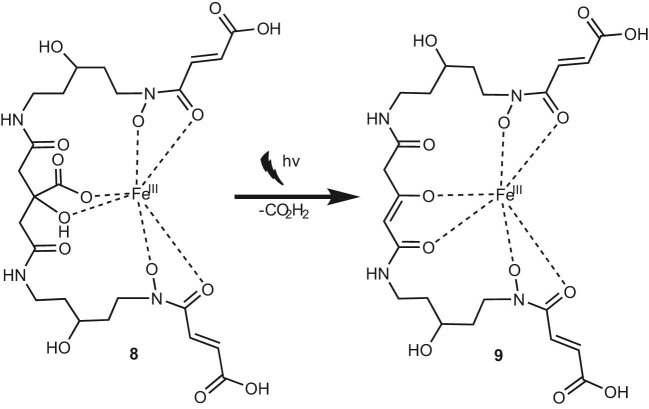
Photolysis reaction of ferric chryseochelin A (**8**) under sunlight leading to the loss of CO_2_H_2_ and photoproduct **9**.

Fe(III)–chryseochelin A (**8**) follows the identical trend as illustrated in Fig. 5. After 3 h of sunlight exposure, a complete conversion of the ferric chryseochelin A to its photoproduct **9** was observed. The mass shift of *m*/*z* 46 from the intact complex (*m*/*z* 674.13726, C_24_H_34_N_4_O_15_Fe^+^, −1.1 ppm) to the photoproduct (*m*/*z* 628.13146, C_23_H_32_N_4_O_13_Fe^+^, −0.7 ppm) was demonstrated by ESI–MS (see Supplementary Fig. S12). Additionally, UV/VIS revealed typical changes such as the appearance of a new absorption maximum at 259 nm, which is characteristic for citrate-derived photoproducts as reported for aerobactin (see Supplementary Fig. S13).^42^ This provides an additional evidence for the incorporated citrate moiety.

As our *Chryseobacterium* strains grow in the light-protected rhizosphere of plants, photoproduct **9** is unlikely to occur naturally and is thought to play a negligible role in biological processes. This is different for bacteria growing in marine environments that are exposed to sun light. For these organisms, ecological and evolutionary pressures must have been involved in favoring photolabile siderophores such as vibrioferrin or petrobactin.^41^

### MS/MS investigation of chryseochelin B

**Fig. 6:**
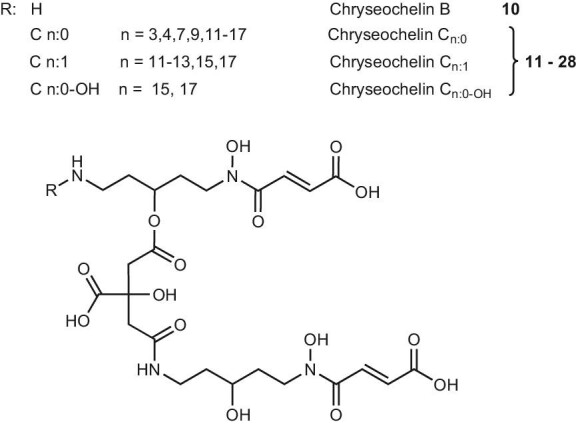
Proposed structures of chryseochelin B (**10**) and its fatty acid derivative chryseochelin C (**11**–**28**) containing various fatty acid groups. For chryseochelin C, the exact numbering is as follows: C n:0 = 3 (**11**), 4 (**12**), 7 (**13**), 9 (**14**), 11 (**15**), 12 (**16**), 13 (**17**), 14 (**18**), 15 (**19**), 16 (**20**), 17 (**21**); C n:1 = 11 (**22**), 12 (**23**), 13 (**24**), 15 (**25**), 17 (**26**), C n:0-OH = 15 (**27**), 17 (**28**).

In the bacterial supernatant, we also identified chryseochelin B (**10**, *R_t_*_ _= 2.83 min, Fig. 6), a second siderophore whose [M + H]^+^ ion has an *m*/*z* value of 621.22430. This *m*/*z* value led to the identical molecular formula C_24_H_36_N_4_O_15_ (1.1 ppm). We wanted to know whether chryseochelin B was a simple stereoisomer of chryseochelin A or whether it was a new siderophore. Several attempts were made to isolate the isobaric chryseochelin B, but the compound degraded during the purification process.

Therefore, a high-resolution MS/MS spectrum (Supplementary Fig. S14) of chryseochelin B was recorded and compared with the MS/MS spectrum of chryseochelin A, whose complete structure was elucidated. At first glance, a very similar spectrum was obtained: The main fragment pair *m*/*z* 233.11327 (C_9_H_17_N_2_O_5_^+^, −0.3 ppm) and 389.11884 (C_15_H_21_N_2_O_10_^+^, −0.6 ppm) are present as well as the fumaric acid fragment (*m*/*z* 99.00764, C_4_H_3_O_3_^+^, 0.3 ppm) and its counter fragment (*m*/*z* 523.22306, C_20_H_35_N_4_O_12_^+^, 2.9 ppm). Also, frequent H_2_O (−*m*/*z* 18) and CO_2_H_2_ (−*m*/*z* 46) losses are observed. At *m*/*z* 100.07587 (C_5_H_10_NO^+^, −1.8 ppm) and 117.10239 (C_5_H_13_N_2_O^+^, −1.3 ppm), characteristic fragments of the 3-hydroxycadaverine substructure are seen. In fact, all detected fragments were similar to those observed for chryseochelin A, except for a single fragment at *m*/*z* 407.12982 (C_15_H_23_N_2_O_11_^+^, −0.4 ppm). This observation can only be explained if chryseochelin B is built from the same units but has a different connectivity: One citrate appendage must be attached by the hydroxy group of the 3-hydroxycadaverine unit. Another indicator of the presence of this ester bond is given by the fragment distribution: While chryseochelin A has a broad and regular fragment distribution due to the presence of only amide bonds, the MS/MS spectrum of chryseochelin B is dominated by the fragments at *m*/*z* 389 and 215, as ester bonds are more prone to fragmentation, as has been reported in several cases.^43^,^44^ Furthermore, the primary amine of chryseochelin B is a good nucleophile that potentially reacts with ketones, aldehydes or esters, justifying why its isolation was not possible.

### Chryseochelin C: fatty acid modifications

**Fig. 7:**
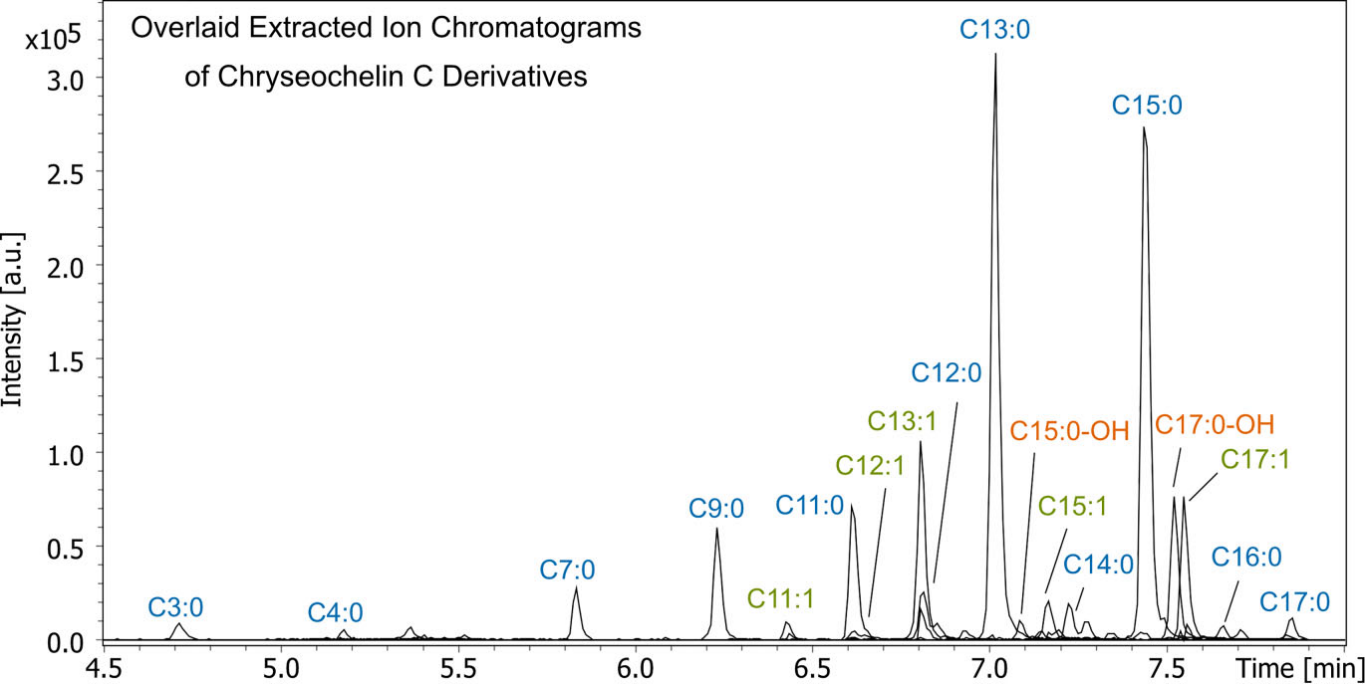
Overlaid extracted ion chromatograms of chryseochelin C (**11**–**28**) derivatives owning various fatty acid chain lengths detected in *Chyseobacterium* supernatant. Compound numbering, retention times and exact masses are available in Supplementary Table S2.

Based on the structural elucidation of chryseochelin A and chryseochelin B by MS/MS, the core structures of chryseochelin C (**11**–**28**) were identified and deducted by mass spectrometric methods (Fig. 6). Chryseochelin C derivatives are obtained by acetylation of the primary amine of chryseochelin B. This leads to the attachment of a saturated, unsaturated, or hydroxylated fatty acid chain of varying length, a common modification in amphiphilic siderophores.^10^ As the citrate and the hydroxamate units remain intact, these derivatives are also capable of binding iron. Retention times and exact masses are given in the Supplementary Table S2. Extracted ion chromatograms of chryseochelin C derivatives are presented in Fig. 7. There it can be clearly seen that odd numbered carbon chains and saturated fatty acid attachments are produced in higher quantities than even or unsaturated/hydroxylated ones.

The identification of these compounds is illustrated by the example of chryseochelin C_15:0_ (**19**). Fragmenting the precursor mass at *m*/*z* 845.43904 (C_39_H_65_N_4_O_16_^+^, 0.1 ppm) at a fixed collision energy of 35 eV gives the spectrum shown in Supplementary Fig. S15A with a dominant fragment containing the fatty acid chain at *m*/*z* 439.31564 (C_24_H_43_N_2_O_5_^+^, −2.3 ppm). The fragment ions at *m*/*z* 233.11344 (C_9_H_17_N_2_O_5_^+^, 1.0 ppm), 389.11861 (C_15_H_21_N_2_O_10_^+^, −1.2 ppm) and 613.33368 (C_30_H_49_N_2_O_11_^+^, 1.0 ppm) are detected with lower intensity. Chryseochelin C_15:0_ also gives a signal at *m*/*z* 621.22347 (loss of fatty acid, C_24_H_37_N_4_O_15_^+^, −2.5 ppm) and at *m*/*z* 747.43990 (loss of fumaric acid, C_35_H_63_N_4_O_13_^+^, 1.7 ppm), but these show higher signal intensity when stepped collision energies are applied (see Supplementary Fig. S14). Additional fragment ions comparable to chryseochelin A are also obtained in the lower mass range (see Supplementary Fig. S16). Due to the presence of the fragment *m*/*z* 407.13065 (C_15_H_23_N_2_O_11_^+^, 2.5 ppm) and the unequal fragment ion distribution as already encountered for chryseochelin B, we assume that the main frameworks of chryseochelin B and C_15:0_ are identical. Further arguments in favor of the hypothesis that only one appendage is attached to the citrate moiety via an ester bond are the following: No doubly acetylated chryseochelin derivatives were detected, which would be possible if the main framework of chryseochelin C had two derivatizable primary amines. Also, no isobaric compounds of chryseochelin C derivatives were found, which would indicate a mixture of constitutional isomers, as in the case for chryseochelin A and chryseochelin B. And last, in the MS/MS spectrum of chryseochelin C_15:0_, a theoretical fragment at *m*/*z* 631.34365 (C_30_H_51_N_2_O_12_^+^) would be expected, if two ester bonds were present. However, this fragment was not found.

In a next step, the connectivity of the fatty acid chain to the main body had to be determined. To rule out the possibility that the fatty acid chain is attached to a carbon and to prove the presence of nine exchangeable protons, an MS/MS spectrum of chryseochelin C_15:0_ in D_2_O was measured (see Supplementary Fig. S17). Furthermore, an MS^3^ spectrum of the *m*/*z* 439 ions containing the fatty acid chain was measured at a collision energy of 25 eV (Supplementary Fig. S15B). For this, the *m*/*z* 439 ions were generated by in-source collision-induced dissociation at 100 eV. At *m*/*z* 225.22191 (C_15_H_29_O^+^, 2.8 ppm), we find a small signal of the ionized fatty acid side chain. Fragments containing nitrogen (*m*/*z* 242.24768, C_15_H_32_NO^+^, 0.7 ppm), the five-membered carbon chain (*m*/*z* 308.29417, C_20_H_38_NO^+^, −2.0 ppm) and the hydroxylamine (*m*/*z* 341.31583, C_20_H_41_N_2_O_2_^+^, −1.2 ppm) were also detected.

This interpretation allowed the elucidation of the connectivity of the fatty acid unit. Similar fragmentation spectra containing characteristic fragments of chryseochelin A were obtained for all other fatty acid containing compounds (Supplementary Fig. S18).

### Growth-inhibiting activity of chryseochelin A against *R. Solanacearum*

**Fig. 8:**
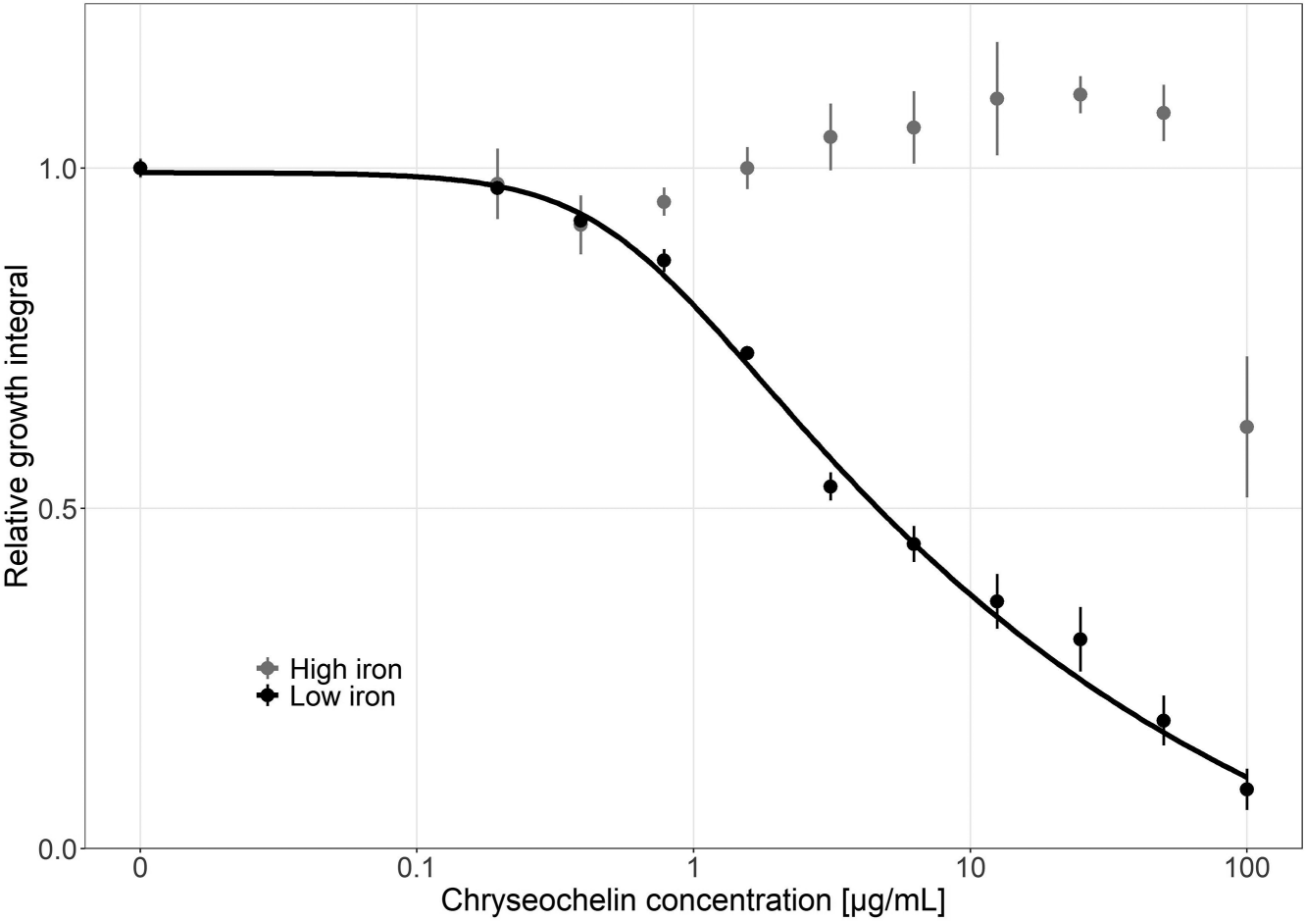
Chryseochelin A (**7**) dose–response curves for Ralstonia *solanacearum* under high and low iron conditions. The plant pathogen *R. solanacearum* was exposed to a concentration gradient of the siderophore chryseochelin A produced by *Chryseobacterium* spp. over a period of 3 days. Values show the growth of the pathogen under chryseochelin A exposure, expressed relative to the growth without exposure in either high (grey) or low (black) iron conditions. Relative growth <1 indicate inhibition. Dots and error bars show mean values and standard errors, respectively, across a minimum of four replicates per concentration. The dose–response curve for the low iron condition was fitted using a four-parameter logistic regression. No dose–response curve could be fitted to the growth data under high iron conditions because of a non-monotonic trajectory.

Several previous studies describe a plant-growth promoting or anti-pathogenic effect of siderophore-producing *Chryseobacteria*.^22–24^,^27^ One study conducted experiments with supernatants of the three *Chryseobacterium* strains analysed here and found that their siderophores can suppress the growth of the plant pathogen *R. solanacearum*.^26^ Here, this hypothesis was tested by exposing *R. solanacearum* to a concentration gradient of purified chryseochelin A in CAA media with low and high iron availability. Under low-iron conditions, a steady decrease in *R. solanacearum* growth was observed with increasing chryseochelin A concentration (Fig. 8). At the highest concentration of 100 μg/mL, a growth reduction of 88.45% was determined compared to CAA media without chryseochelin, and the model yielded an IC50-value (growth reduced by 50%) of 4.64 μg/mL. In contrast, no growth inhibition was observed under high iron conditions (50 μM FeCl_3_), except for the highest concentration of chryseochelin A. These results suggest that chryseochelin A has no antibiotic properties per se, but can strongly bind the residual iron in the growth medium, thereby inducing iron starvation and growth arrest in *R. solanacearum*.

Under high iron conditions, the growth of *R. solanacearum* was promoted at intermediate chryseochelin A concentrations (12.5 μg/mL). This effect could be explained by the fact that iron is toxic at high concentrations. Thus, at intermediate concentrations, chryseochelin A can detoxify the environment by binding iron, while still leaving enough iron available to promote *R. solanacearum* growth.

Taken together, these assays demonstrate the growth-suppressing activity of chryseochelin A, which can serve as the basis for future studies to extend activity tests to other pathogens and potential applications in the field.

### Classification of chryseochelin siderophores

So far, siderophores produced by *Chryseobacterium* species remained chemically uncharacterized. From the phylum *Bacteroidota*, to which the genus *Chryseobacterium* belongs, only the siderophores of *Tenacibaculum* sp. A4K-17^45^ (tenacibactins) and of *Fulvivirga* sp. W222^46^ (fulvivirgamides) have been elucidated. These siderophores consist of several cadaverine units analogous to chryseochelin, suggesting that this substructure might be conserved within the phylum. In contrast to this, the citrate and fumaric acid moiety of chryseochelin cannot be found in tenacibactins and fulvivirgamides.

Other citrate-derived siderophores, which are chemically related to chryseochelins, are produced by various marine and terrestrial bacteria.^10^ Cadaverine units are also found in these, e.g. in nannochelin A (**5**) or arthrobactin (**3**). However, the hydroxylation at the third position of the cadaverine unit is unique for chryseochelin. As for the hydroxamate unit, a conjugated double bond is often seen, as in acinetoferrin (**2**), but no case is known where it is directly followed by a carboxylic acid. Both the hydroxy group and the fumaric acid make chryseochelin A considerably more polar than other siderophores and increase solubility.

**Fig. 9:**
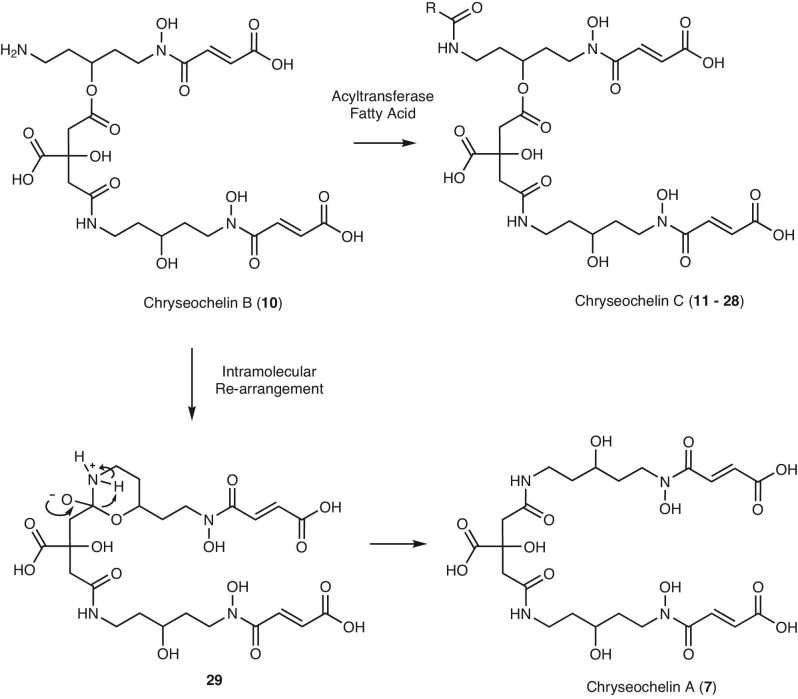
Proposed biotransformation of chryseochelin B (**10**) into chryseochelin A (**7**) and C (**11**–**28**).

In addition to chryseochelin A, we were able to identify chryseochelin B by mass spectrometric means. While chryseochelin A is stable and can be isolated from the bacterial supernatant, chryseochelin B decomposes due the presence of a reactive primary amine. This primary amine is a result of the connection of one cadaverine unit to the citrate moiety via an ester bond. The question arose whether similar cases had already been reported in the literature. Indeed, a striking similarity can be found to achromobactin (**6**) (Fig. 1) which is produced by the soft rot plant pathogen *Dickeya dadantii* and which depletes infected plants of iron.^10^ To the best of our knowledge, achromobactin is the only other citrate-based siderophore that incorporates an ester bond. The lability of chryseochelin B is easily explained by looking at the biosynthetic pathway of achromobactin (Supplementary Fig. S19A).^18^ Briefly described, serine and 2,4-diaminobutyric acid are attached to a citrate molecule by various enzymes via an ester and amide bond, respectively. Afterward, an α-ketoglutaric acid reacts with the primary amine groups and is further modified, leading to the formation of achromobactin. This last conversion occurs very rapidly because the intermediate, which contains a free amine group and an ester bond, is known to be unstable as an intramolecular rearrangement is possible via a five-membered ring (Supplementary Fig. S19B).^47^ Transferring this knowledge to chryseochelin B, the following hypothetical biotransformations could take place, as illustrated in Fig. 9. If the primary amine remains underivatized, the molecule will rearrange intramolecularly via an energetically favored six-ring configuration (**29**) to chryseochelin A, whose stability is higher due to the formation of a new amide bond. On the other hand, the primary amine can also serve as a derivatization point and be protected by fatty acids. In this way, *Chryseobacterium* spp. can produce chryseochelin C. The hypothesis that chryseochelin B is indeed the biological precursor is supported by our observation that chryseochelin B disappears after incubation times longer than 48 h, while chryseochelin A and C accumulate (data not shown). However, further studies are needed to fully understand and prove the biosynthetic pathway of chryseochelins.

Finally, the amphiphilic properties of chryseochelin C with its fatty acid chain need to be addressed. Overall, only a few amphiphilic siderophores such as rhizobactin 1021, ochrobactin, mycobactin, and aquachelin are known.^10^,^48^,^49^ The distantly related fulvivirgamides from Fulvivirga sp. W222 are also found in derivatized form with fatty acids.^46^ However, most of these compounds are produced by marine bacteria. The lipophilic tail is thought to affect membrane affinity and prevent diffusion in sea water.^50^,^51^ Yet, little is known about the strategic advantage of such fatty acid appendages in soil. One explanation could be that *Chryseobacterium* spp. follow a dual strategy for iron scavenging, one operating via the diffusible chryseochelin A to obtain iron from more distant sources, and one operating via the membrane-embedded chryseochelin C to scavenge iron that is in the immediate vicinity of the cell.^9^,^52^

## Conclusions

We report here the complete structure and characterization of chryseochelin A, a citrate-based siderophore from rhizobacterial *Chryseobacterium* strains, which showed growth-inhibitory properties against the plant-pathogenic strain *R. solanacearum*. Two novel substructures (3-hydroxycadaverine and fumaric acid) were found that have not been reported before. We further present uncommon chryseochelin derivatives containing ester connectivity (chryseochelin B) and saturated, unsaturated and hydroxylated fatty acids (chryseochelin C) whose preliminary structures are based on mass spectroscopic data. We assume that this unique structural diversity of chryseochelins contributes to the survival of *Chryseobacterium* spp. in the rhizosphere as well as to the protection of plants against pathogen invasion. Thus, chryseochelin is an interesting siderophore for agricultural applications as a pathogen control agent or biofertilizer. It can also be used as a model siderophore for basic research in the field of microbiome–plant interactions. Furthermore, studies can be conducted on the biological advantage of the amphiphilic derivatization or the elucidation of the unusual biosynthetic pathway. Because siderophores are also of great importance in the medical field, chryseochelin could be interesting as a potential agent to combat the siderophores secreted by human pathogens during infections or as a cancer drug.^8^,^9^,^53^

## Supplementary Material

mfad008_Supplemental_File

## Data Availability

The data underlying this article are available in the article and in its online supplementary material.
