# Supplementary material for: Chryseochelins—structural characterization of novel citrate-based siderophores produced by plant protecting Chryseobacterium spp
Source: Metallomics. 2023 Feb 15;15(3):mfad008. doi: 10.1093/mtomcs/mfad008 (PMC9989332; doi:10.1093/mtomcs/mfad008)
Supplement: mfad008_Supplemental_File [file mfad008_supplemental_file.pdf]

Supplementary

Chryseochelins – Structural Characterization of Novel Citrate-Based Siderophores Produced by Plant Protecting *Chryseobacterium* spp.

[1]Karoline Rehm, [2]Vera Vollenweider, [3,4]Shaohua Gu, [5]Ville-Petri Friman, [2]Rolf Kümmerli, [6,#] Zhong Wei and [1,#]Laurent Bigler

[1]University of Zurich, Department of Chemistry, Winterthurerstr. 190, 8057 Zurich, Switzerland
[2]University of Zurich, Department of Quantitative Biomedicine, Winterthurerstr. 190, 8057 Zurich, Switzerland
[3] Center for Quantitative Biology, Academy for Advanced Interdisciplinary Studies, Peking University, Beijing, 100871, China
[4]Peking-Tsinghua Center for Life Sciences, Academy for Advanced Interdisciplinary Studies, Peking University, Beijing, 100871, China
[5]University of York, Department of Biology, Wentworth Way, York YO10 5DD, UK
[6]Jiangsu Provincial Key Lab for Organic Solid Waste Utilization, Jiangsu Collaborative Innovation Center for Solid Organic Waste Resource Utilization, National Engineering Research Center for Organic-based Fertilizers, Nanjing Agricultural University, Nanjing, P R China

A) Detailed RP-HPLC Siderophore Purification

Preparative scale RP-HPLC purification was achieved on a Shimadzu HPLC system build from two LC-20AP pumps, a DGU-20A3 degasser, a CBM-20A communication bus module, an SPD-20A UV/VIS detector and a FRC-10A fraction collector. 5 mL of desalted and pre-concentrated bacterial supernatant was injected. A Triat C18 column (150 x 30 mm, 5 µm, YMC-Actus) was used for chromatographic separation at room temperature. H_2O and MeOH were employed as eluent A and B at a flow rate of 40 mL/min. The following gradient was run: (i) 0 – 2 min 5% B; (ii) ramping to 30% B until 15 min; (iii) ramping to 100% B until 30 min; (iv) flushing the column with 100% until 50 min. The fraction collector collected all peaks with an absorption wavelength of 270 nm. The following HPLC chromatogram was obtained of the crude bacterial extract with chryseochelin A eluting around 14 min.

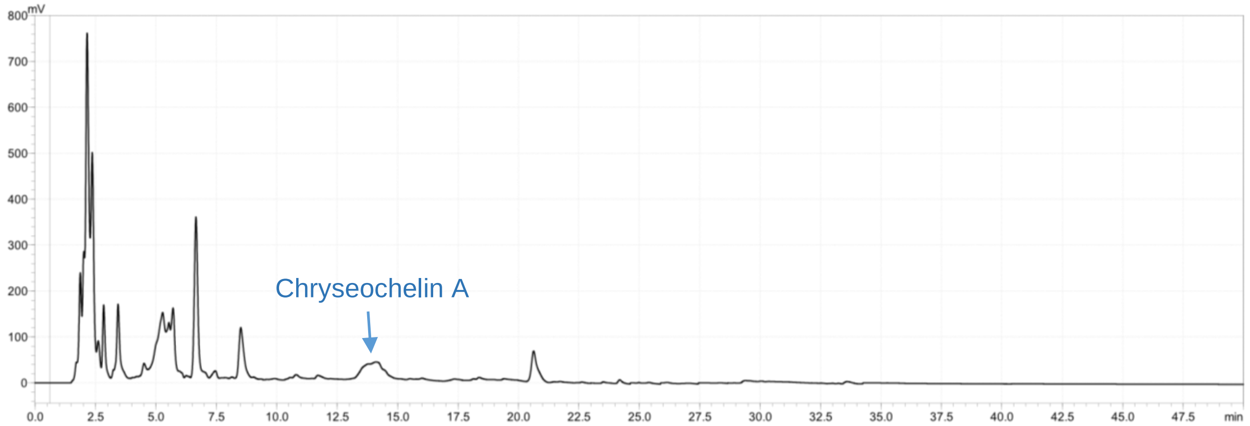

Figure S1. HPLC chromatogramm (270 nm) of the crude bacterial extract and indicated chryseochelin A peak that was collected for the second purification step.

The collected chryseochelin A fraction was lyophilized leaving behind a beige powder. This powder was readily soluble in 25 µL of deionized H_2O and was subjected to a second purification step on an analytical scale RP-HPLC. Analytical scale RP-HPLC purification was performed on a Vanquish Horizon UHPLC System by Thermo Fisher (Waltham, MA, USA) build from a Vanquish binary pump H, a Vanquish split sampler HT, temperature-controllable Vanquish column compartment and the Vanquish diode array detector. Chromatographic separation was achieved at 40°C on an CORTECS C18 column (150 × 4.6 mm, 2.7 µm, Waters). Eluent A consisted of H_2O + 0.1% HCOOH and B of CH_3CN + 0.1% HCOOH. The following gradient was applied at a constant flowrate of 1.0 mL: (i) 4% B isocratic from 0.0-0.5 min; (ii) linear increase to 11% B until 5.25 min; (iii) linear increase to 95% until 7.0 min; (iv) holding 95% B until 10.0 min (vi) back to the starting conditions of 4% B until 10.5 min; (vii) equilibration for 4.5 min until the next run. The absorption wavelength 270 nm was again monitored while fractions were collected by hand. The following HPLC chromatogram was obtained with chryseochelin A eluting at 4.68 min.

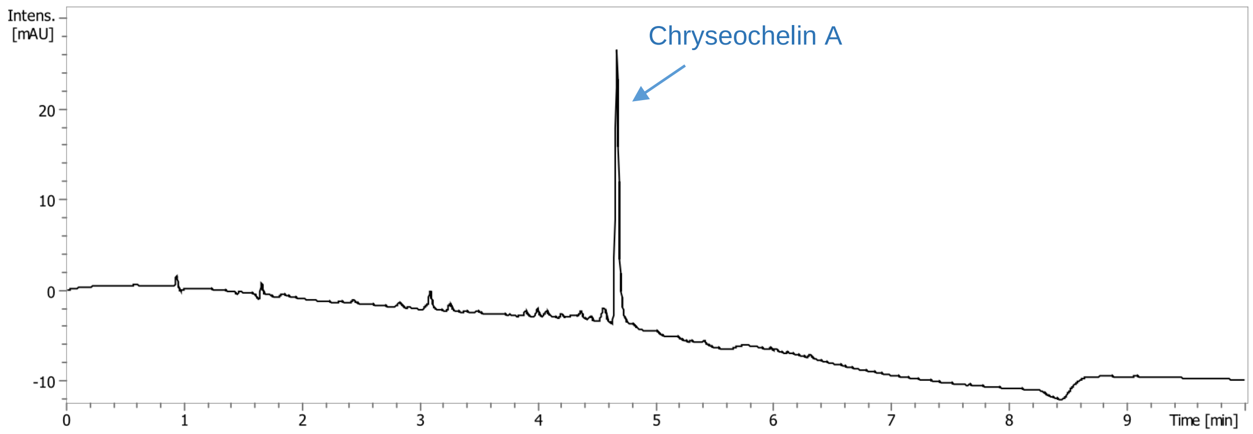

Figure S2. HPLC chromatogramm (270 nm) of chryseochelin A after preparative scale RP-HPLC purification. The chryseochelin A peak was collected for NMR analysis.

The final chryseochelin A fraction was >95% pure according to UV-VIS absorption and LC-MS. It was lyophilized and the white powder (3.1 mg) was stored at −20°C until it was analyzed by NMR.

B) Supplementary Data

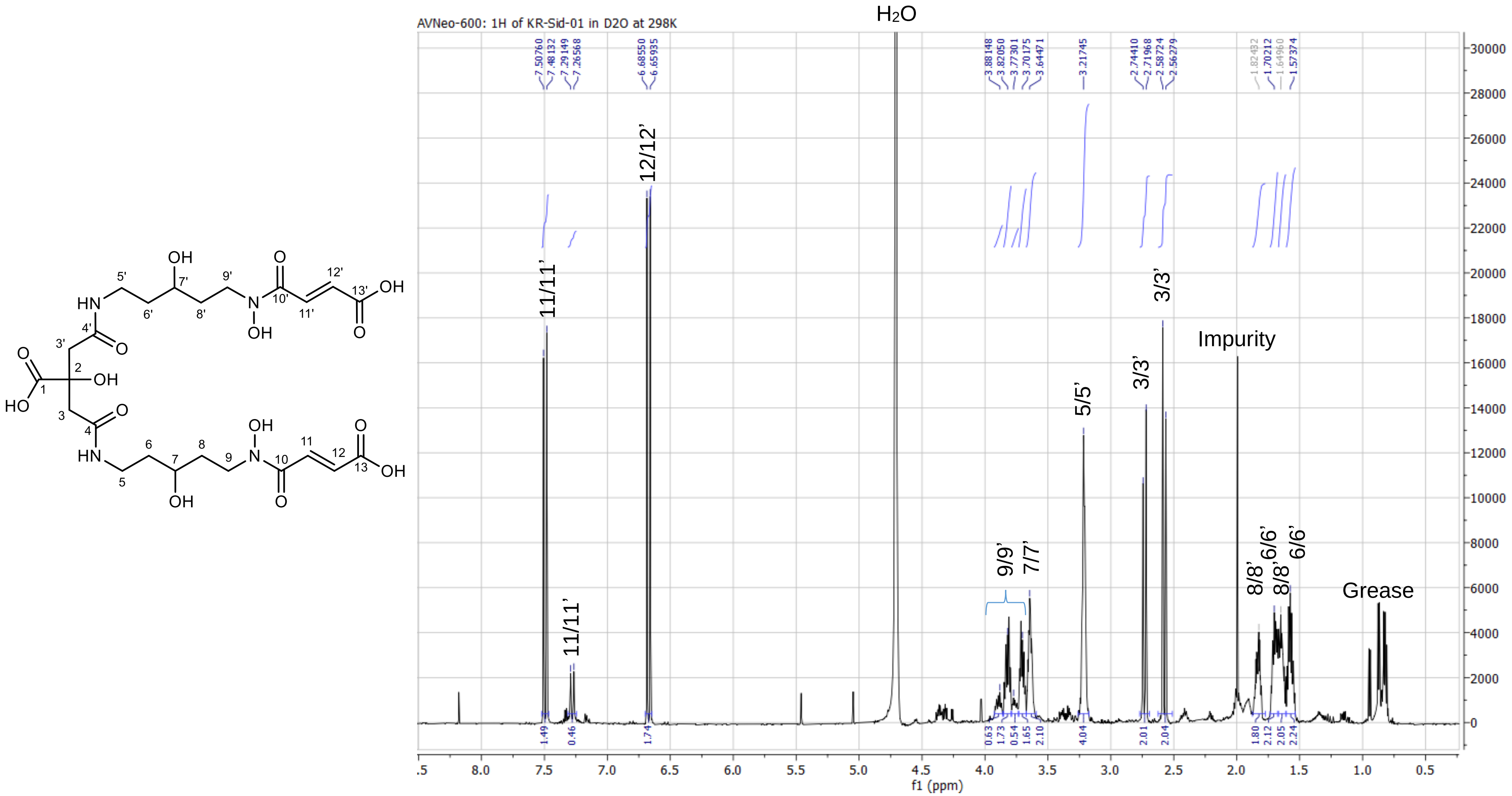

Figure S3. ^{1}H NMR spectrum of chryseochelin A.

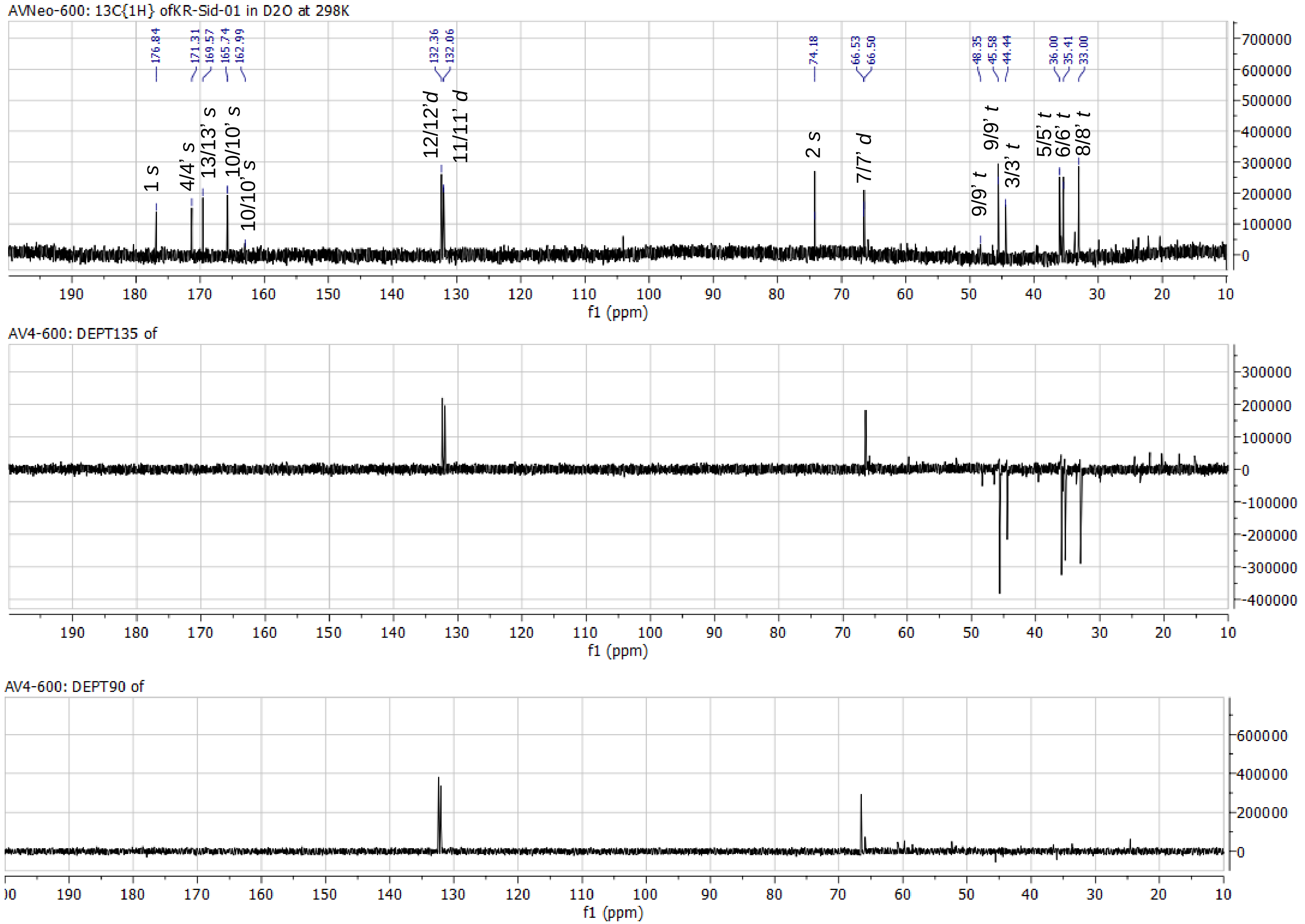

Figure S4. ^{13}C NMR spectrum of chryseochelin A including DEPT 135 and DEPT 90 data.

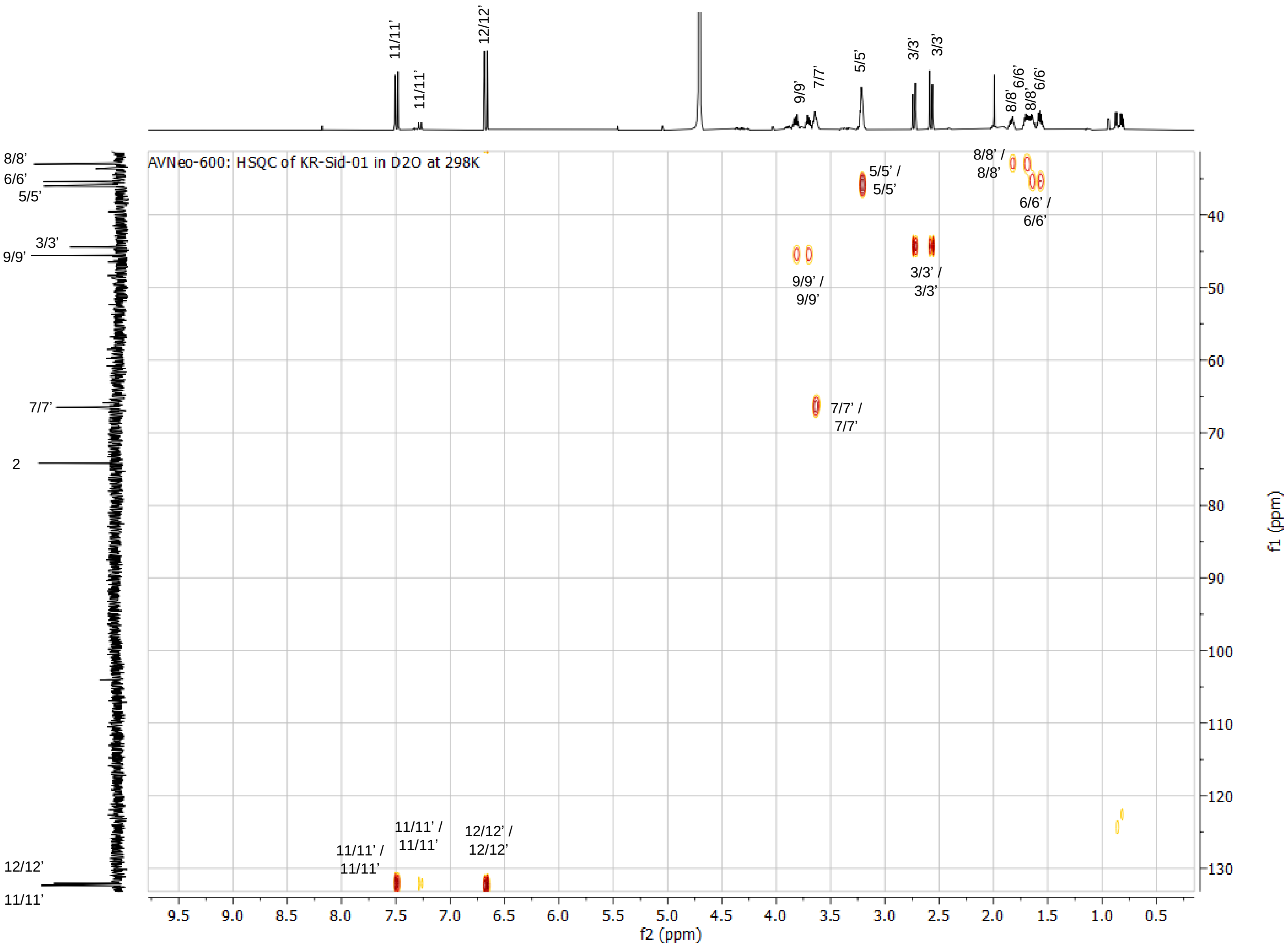

Figure S5. HSQC spectrum of chryseochelin A.

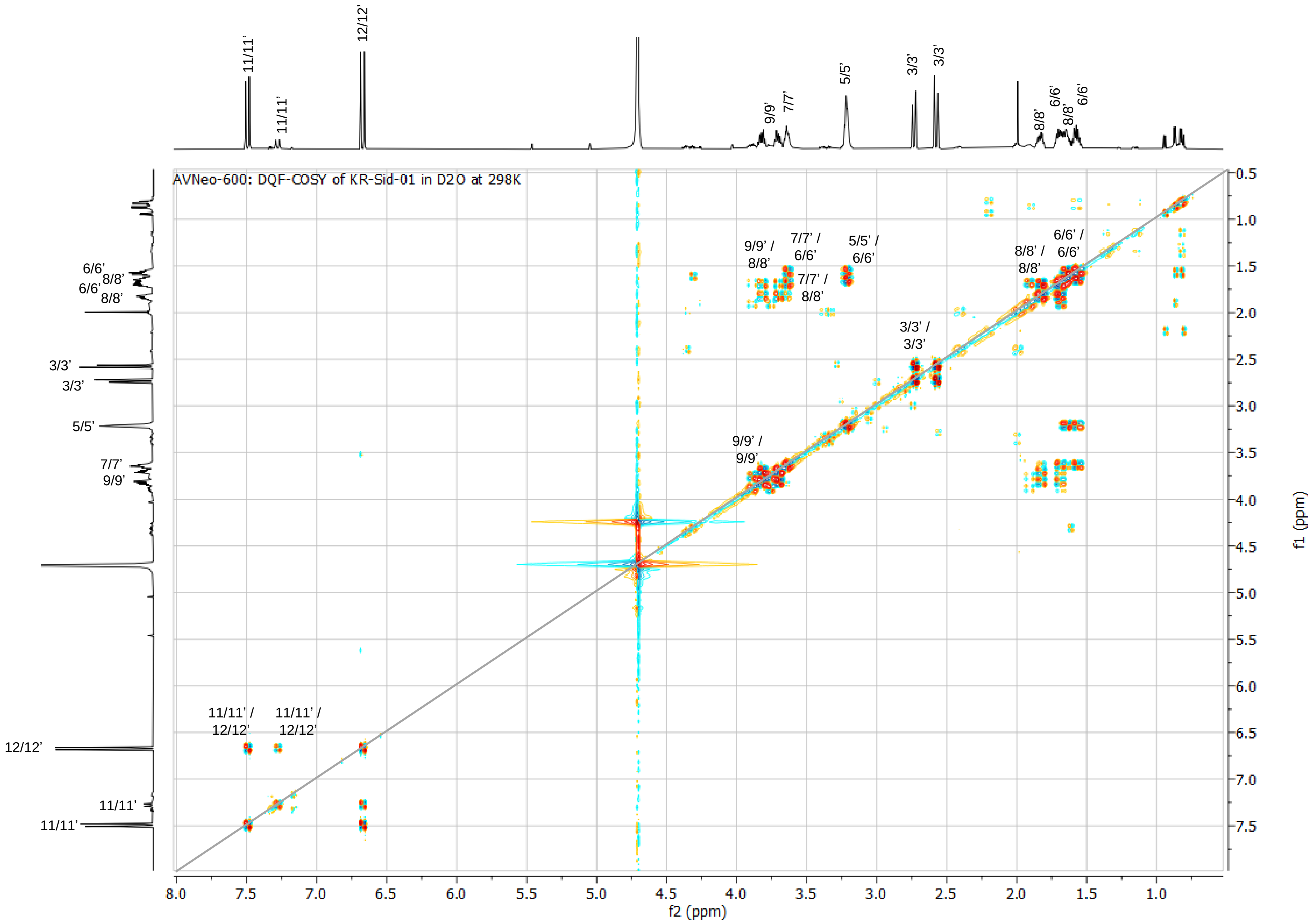

Figure S6. COSY spectrum of chryseochelin A.

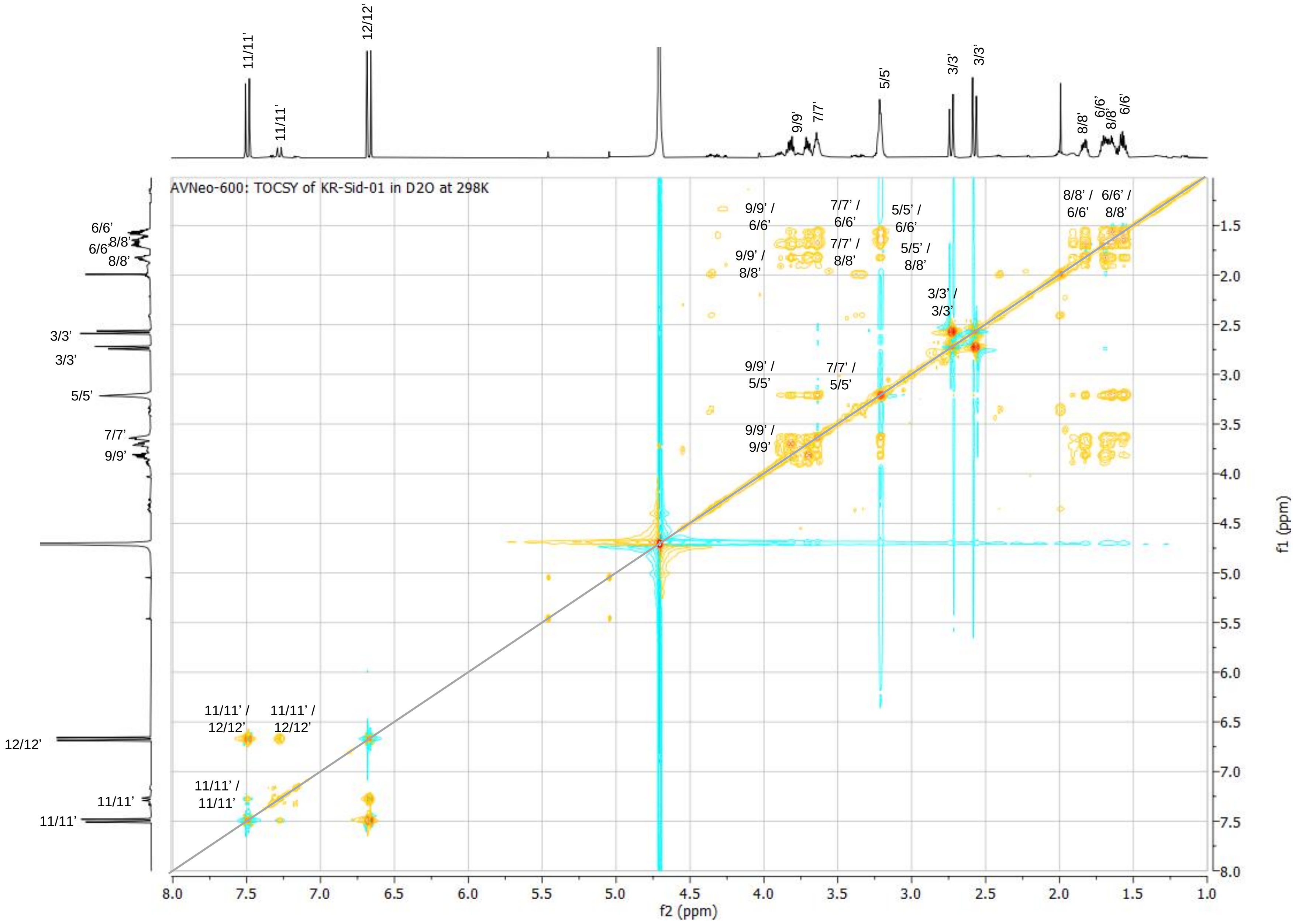

Figure S7. TOCSY spectrum of chryseochelin A.

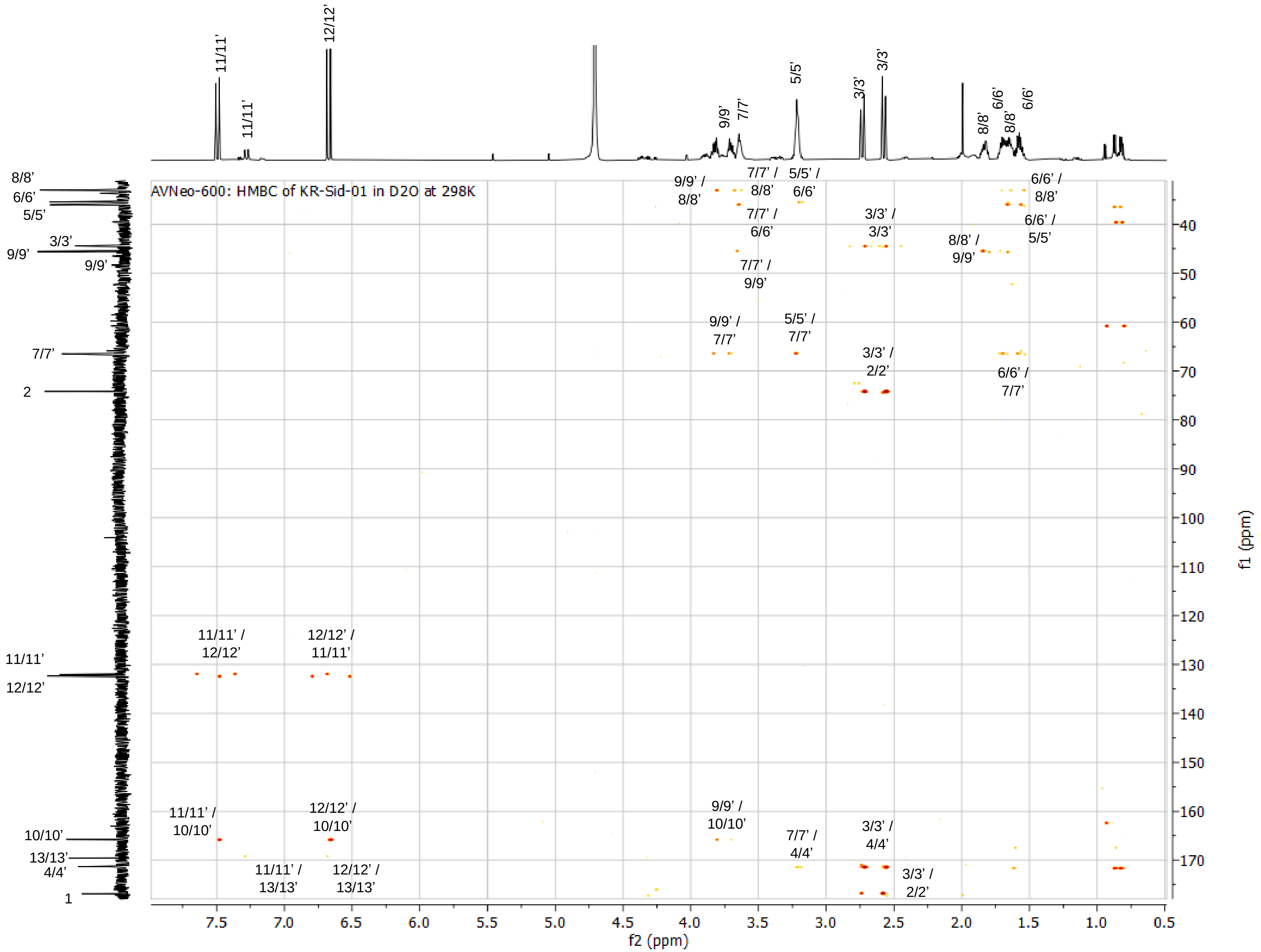

Figure S8. HMBC spectrum of chryseochelin A.

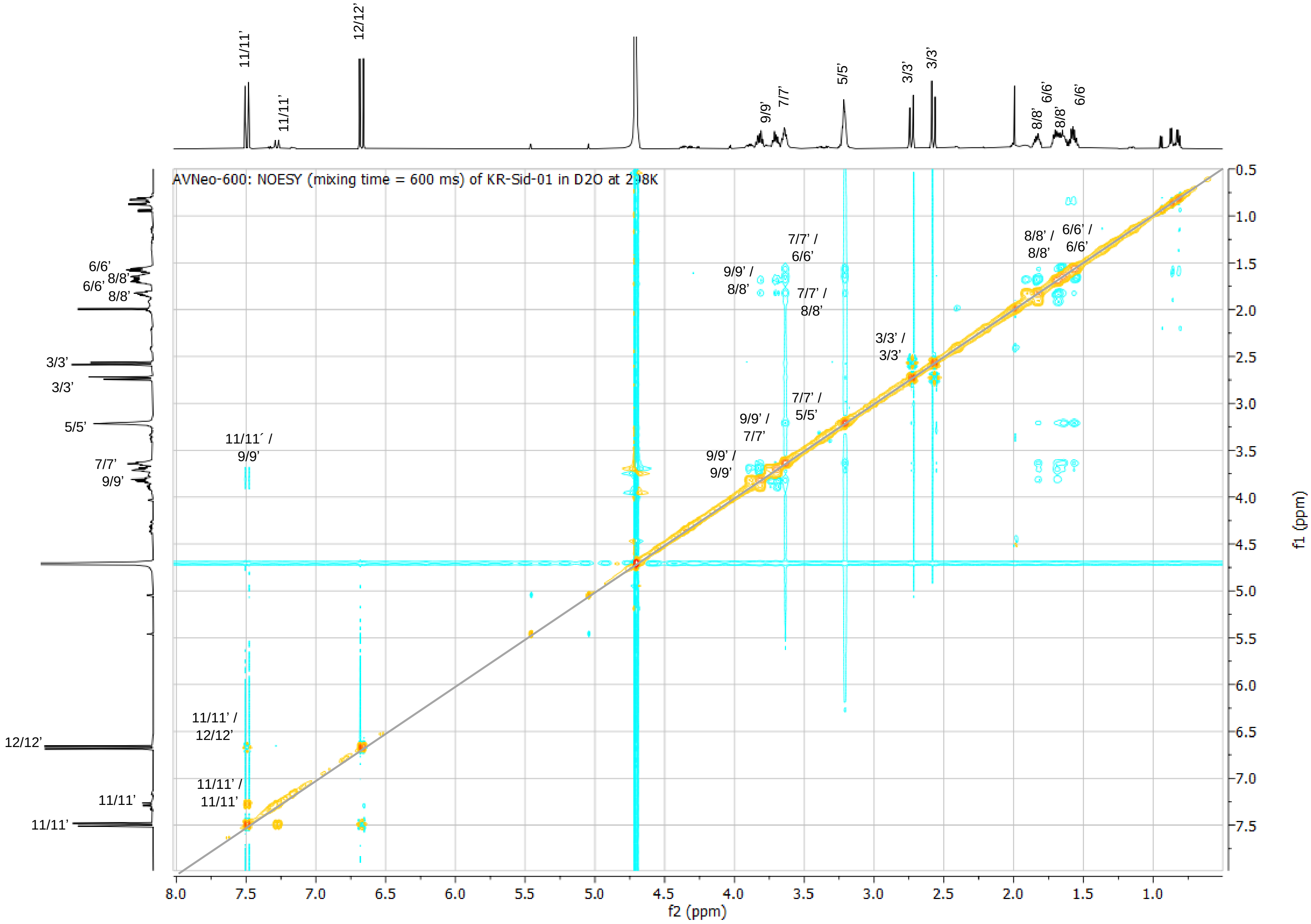

Figure S9. NOESY spectrum of chryseochelin A.

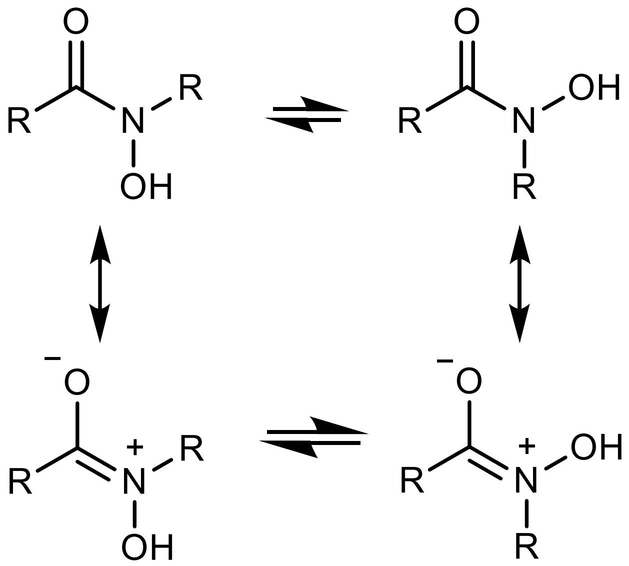

Figure S10. Isomerism of hydroxamates forming amides and iminols in (Z)- and (E)-configuration leading to isomeric proton and carbon pairs in NMR spectra.

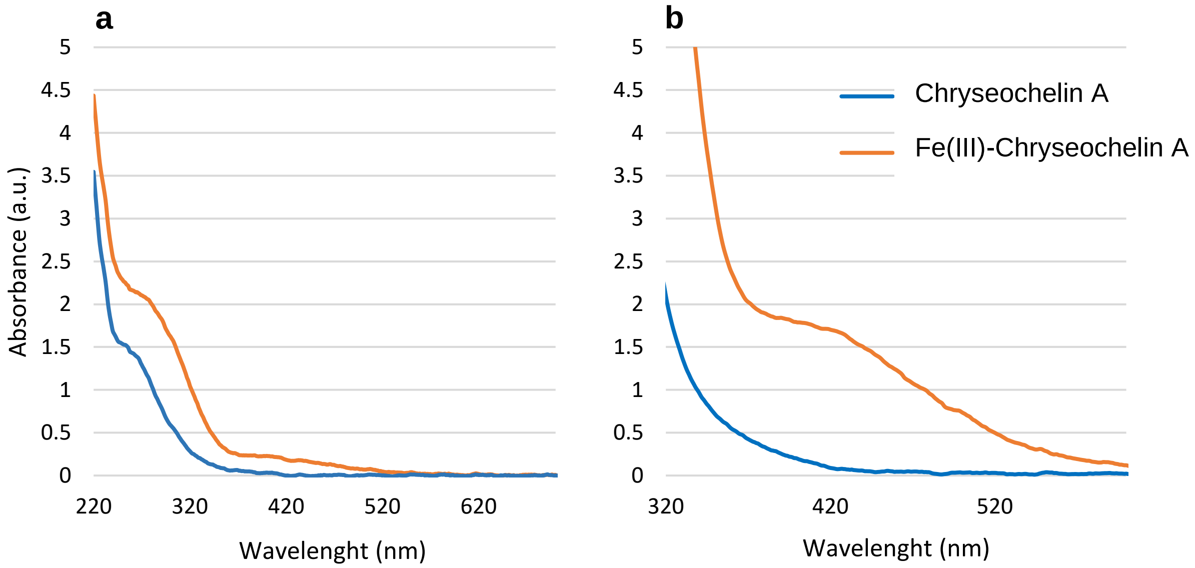

Figure S11. UV/VIS spectra of chryseochelin A and Fe(III)-chryseochelin A at a concentration of (a) 0.2 mM and (b) 2 mM in 20 mM phosphate buffered H_2O (pH 7).

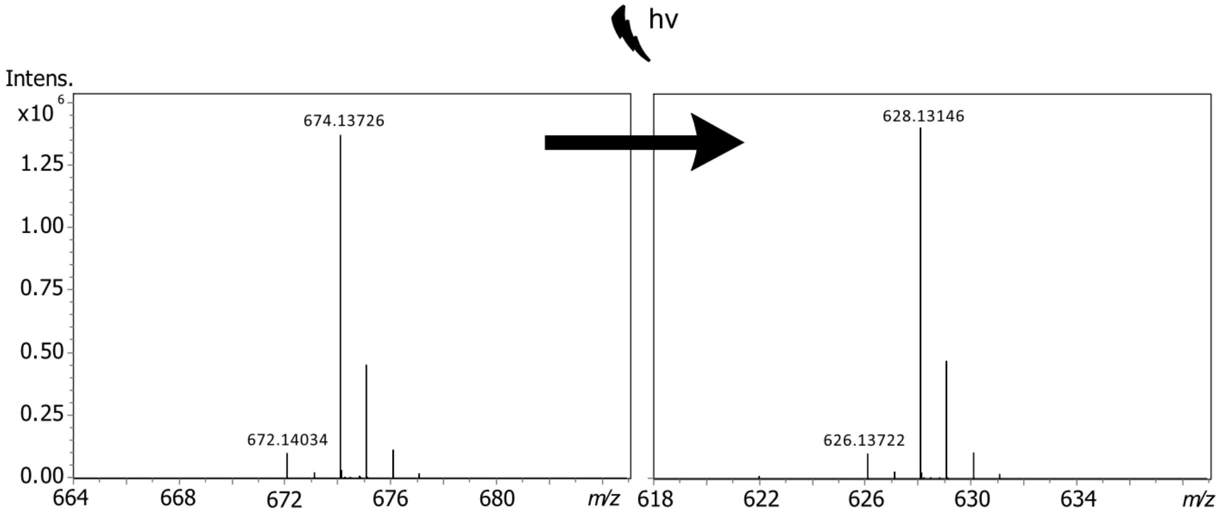

Figure S12. Loss of CO_2 and H_2 of Fe(III)-chryseochelin A (*m/z* 674, $[M+H]^+$) after exposure to sun light leading to the formation of the photoproduct of Fe(III)-chryseochelin A (*m/z* 628, $[M+H]^+$).

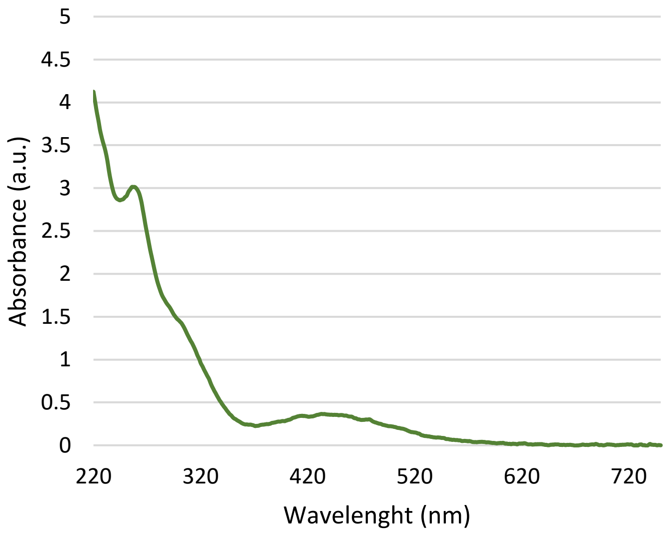

Figure S13. UV/VIS spectra of the photoproduct of Fe(III)-chryseochelin A compley after 3 hours of natural sun exposure at a concentration of 0.2 mM in 20 mM phosphate buffered H_2O (pH 7).

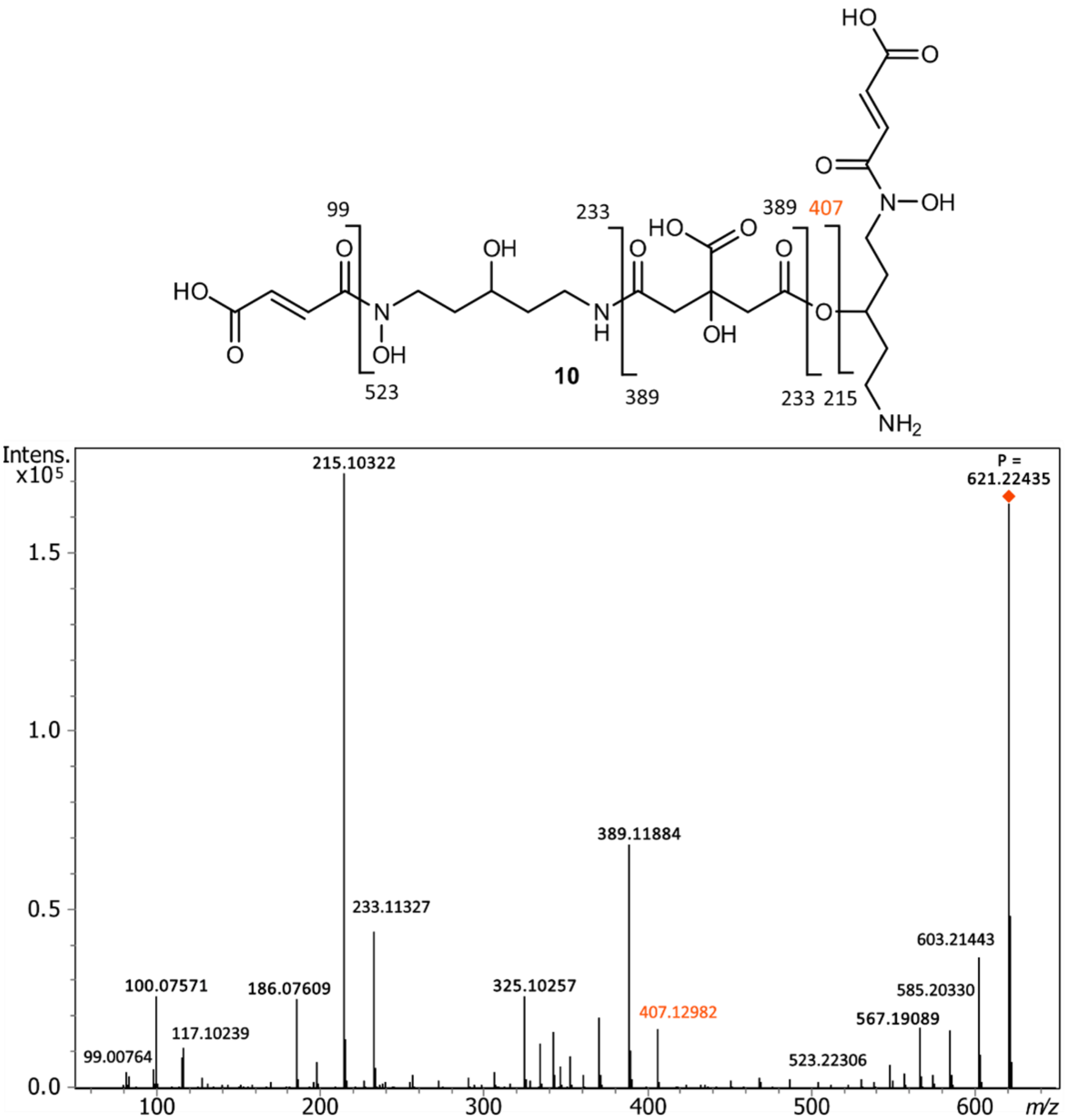

Figure S14. MS/MS spectrum of chryseochelin B (**10**) (precursor ion *m/z* 621) at a collision energy of 35 eV. A peak list is available in Table S1.

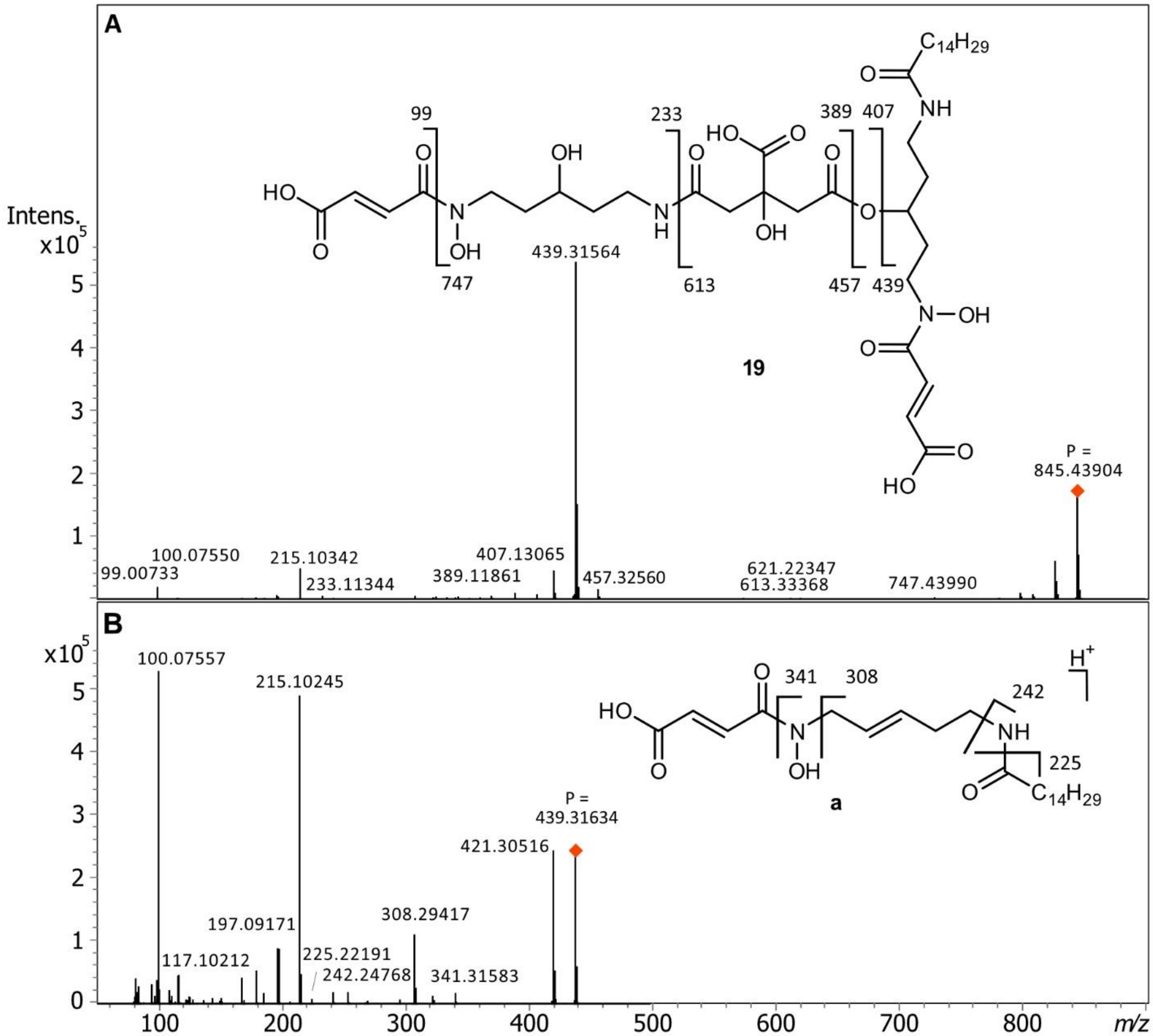

Figure S15. A) MS/MS spectrum of chryseochelin $C_{15:0}$ (**19**) (*m/z* 845) at a collision energy of 35 eV. The peak list of the MS/MS spectrum is available in Table S1. B) MS^3 spectrum of the major chryseochelin $C_{15:0}$ fragment (**a**) (*m/z* 439) generated by isCID at 100 eV and then fragmented at 25 eV.

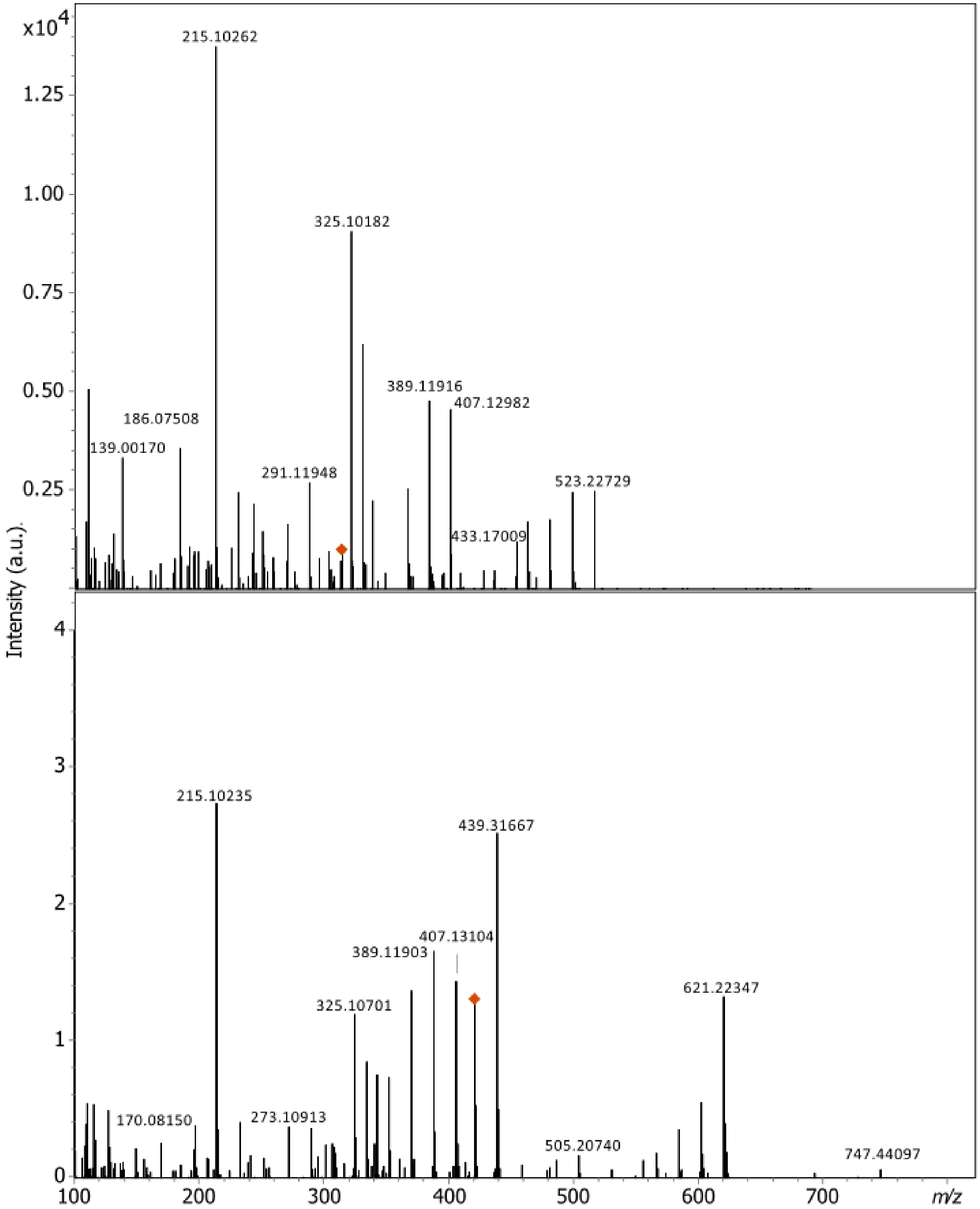

Figure S16. Comparison of the fragment spectra of chryseochelin B (precursor: $[M+2H]^{2+}$, *m/z* 311) (top) and chryseochelin $C_{15:0}$ (precursor: $[M+2H]^{2+}$, *m/z* 423) (bottom) at a stepped collision energy of 20 and 50 eV. Fragmentation precursors are marked in orange.

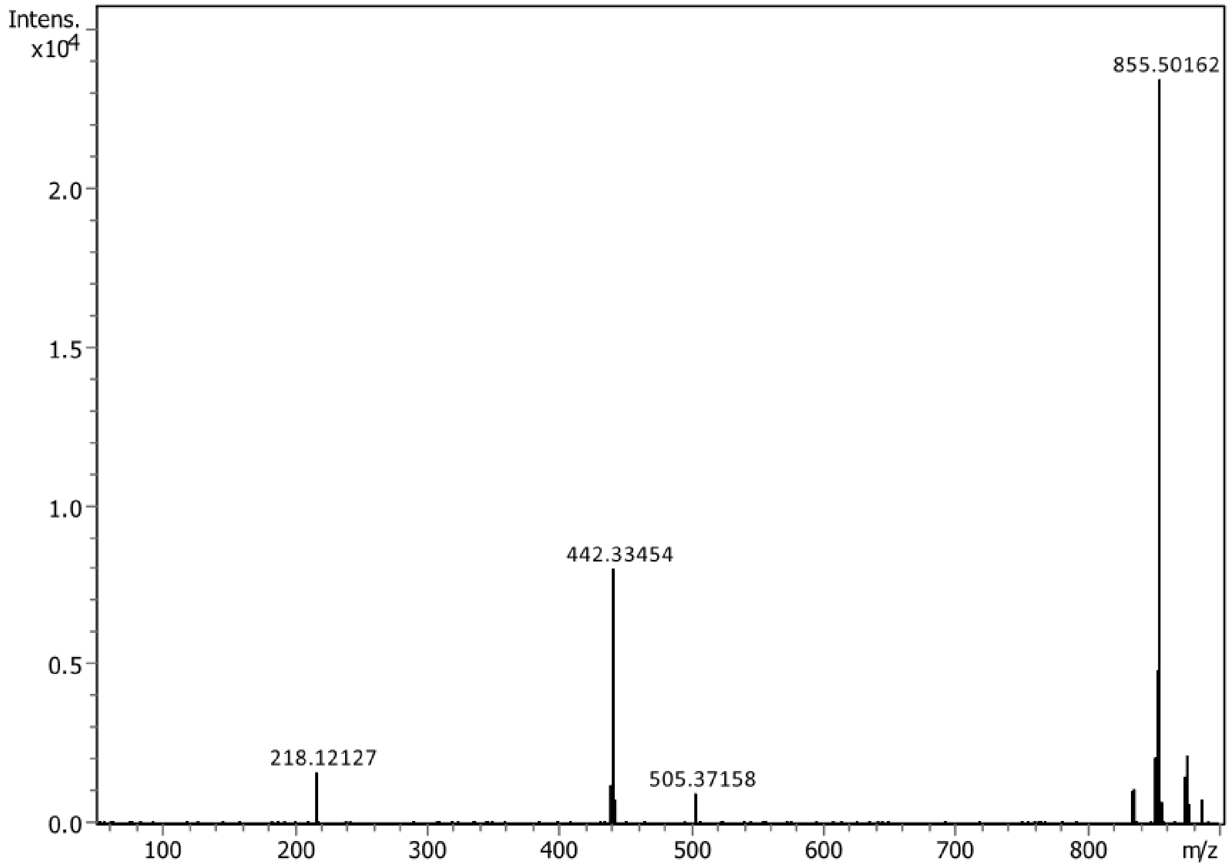

Figure S17. Fragmentation pattern of chryseochelin $C_{15:0}$ in deuterated solvent (precursor: $[d_9\text{-M+D}]^+$, *m/z* 855) at a collision energy of 35 eV showing the presence of 9 exchangeable protons in the in-tact precursor.

Figure S18. Collection of MS/MS spectra of chryseochelin C derivatives obtained at a collision energy of 35 eV.

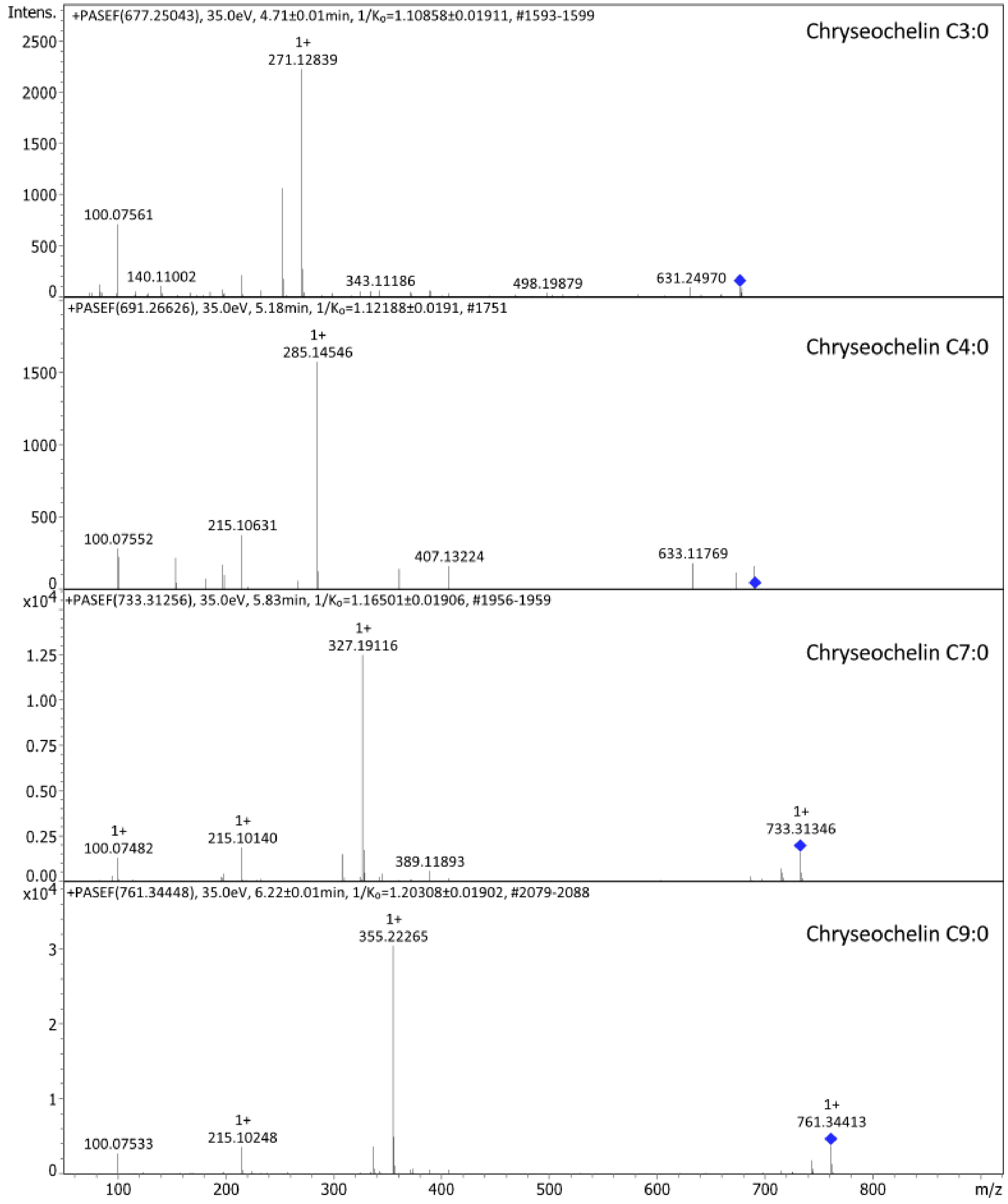

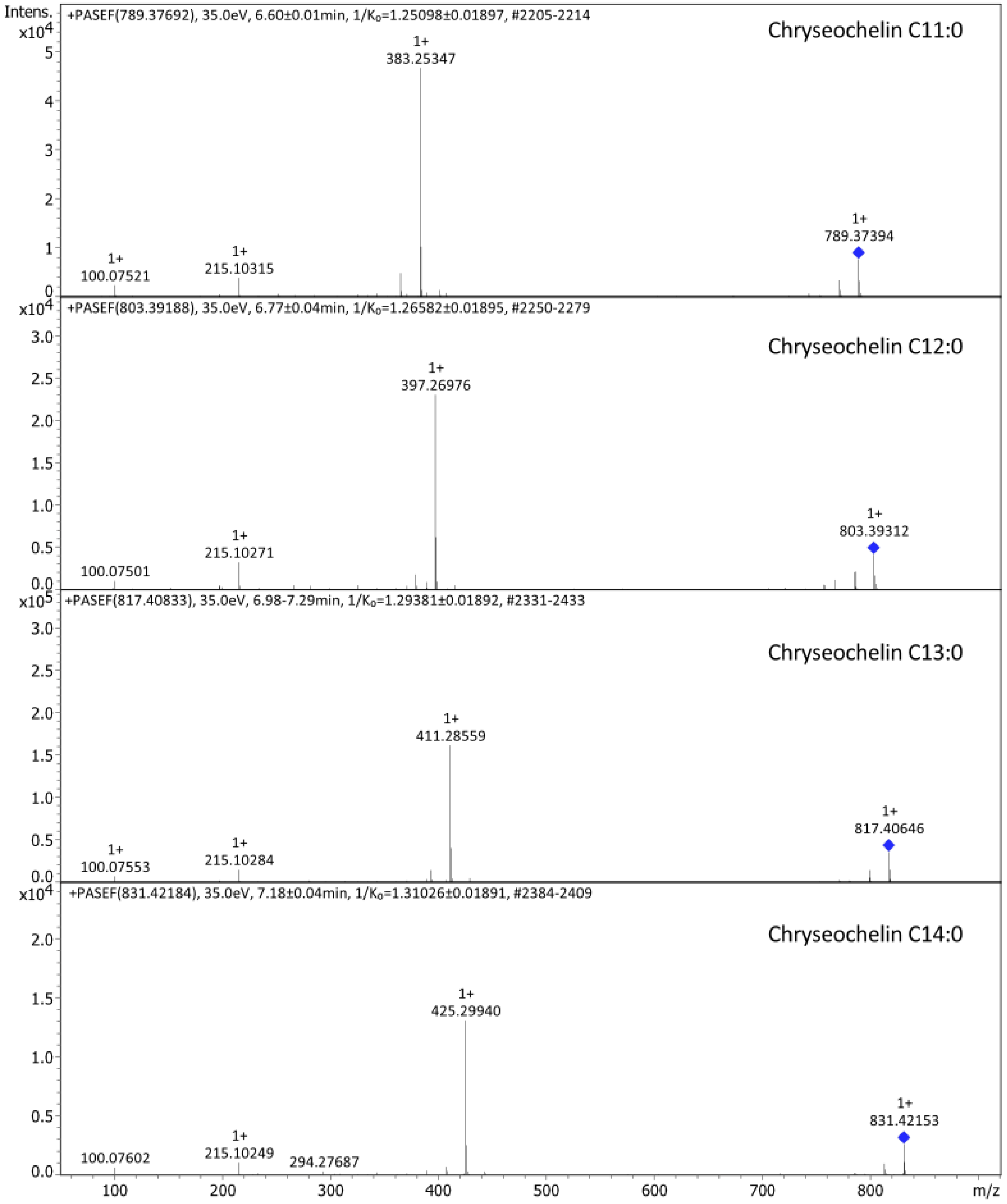

Intens.
x10^4
+PASEF(789.37692), 35.0eV, 6.60±0.01min, 1/K₀=1.25098±0.01897, #2205-2214
Chryseochelin C11:0
1+
383.25347
1+
789.37394
1+
100.07521
1+
215.10315
x10^4
+PASEF(803.39188), 35.0eV, 6.77±0.04min, 1/K₀=1.26582±0.01895, #2250-2279
Chryseochelin C12:0
1+
397.26976
1+
803.39312
100.07501
1+
215.10271
x10^5
+PASEF(817.40833), 35.0eV, 6.98-7.29min, 1/K₀=1.29381±0.01892, #2331-2433
Chryseochelin C13:0
1+
411.28559
1+
817.40646
1+
100.07553
1+
215.10284
x10^4
+PASEF(831.42184), 35.0eV, 7.18±0.04min, 1/K₀=1.31026±0.01891, #2384-2409
Chryseochelin C14:0
1+
425.29940
1+
831.42153
100.07602
1+
215.10249
294.27687
m/z

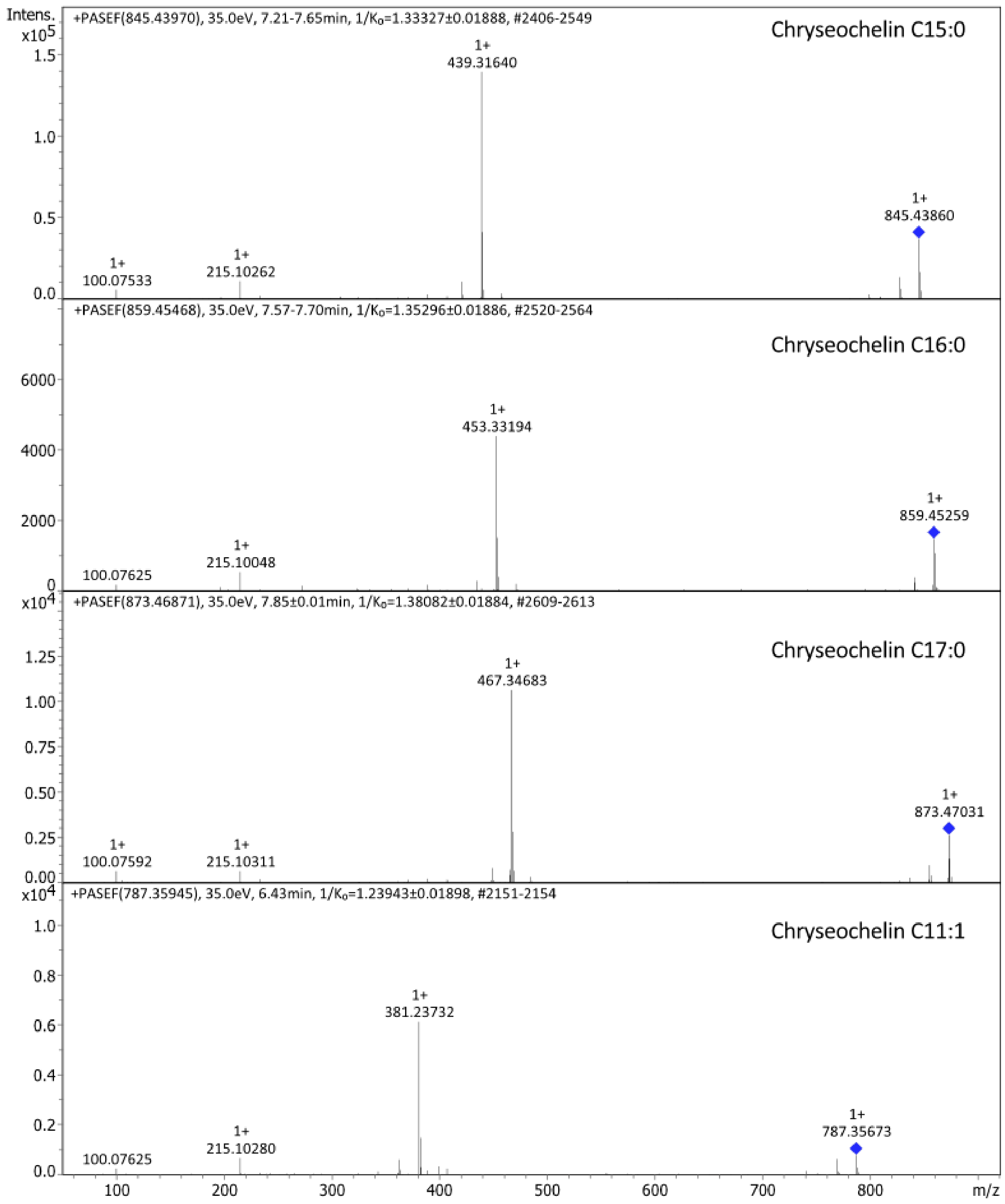

Intens.
x10^5
+PASEF(845.43970), 35.0eV, 7.21-7.65min, 1/K₀=1.33327±0.01888, #2406-2549
Chryseochelin C15:0
1+ 439.31640
1+ 845.43860
1+ 100.07533
1+ 215.10262
+PASEF(859.45468), 35.0eV, 7.57-7.70min, 1/K₀=1.35296±0.01886, #2520-2564
Chryseochelin C16:0
1+ 453.33194
1+ 859.45259
100.07625
1+ 215.10048
x10^4
+PASEF(873.46871), 35.0eV, 7.85±0.01min, 1/K₀=1.38082±0.01884, #2609-2613
Chryseochelin C17:0
1+ 467.34683
1+ 873.47031
1+ 100.07592
1+ 215.10311
x10^4
+PASEF(787.35945), 35.0eV, 6.43min, 1/K₀=1.23943±0.01898, #2151-2154
Chryseochelin C11:1
1+ 381.23732
1+ 787.35673
100.07625
1+ 215.10280
m/z

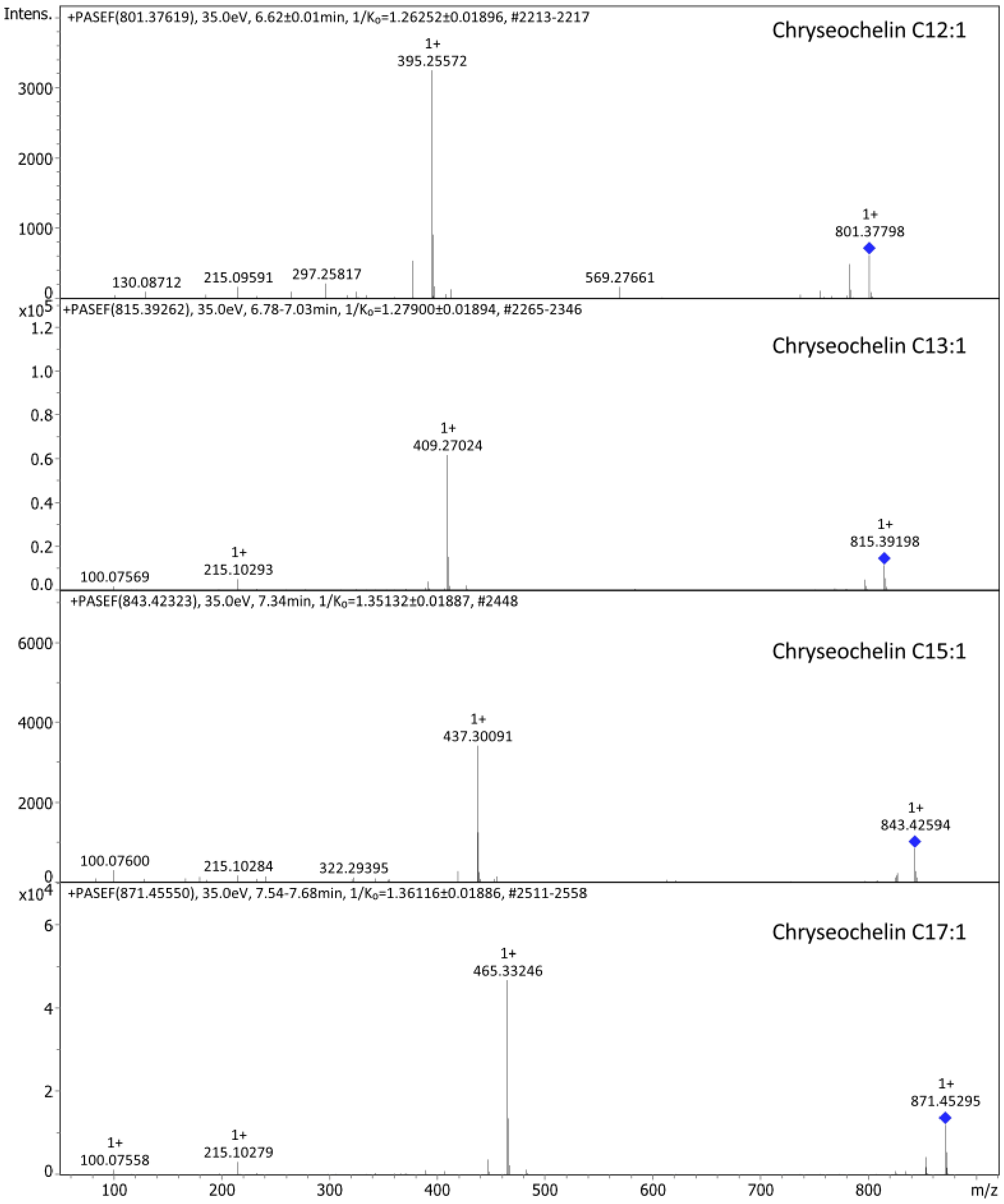
Intens.
+PASEF(801.37619), 35.0eV, 6.62±0.01min, 1/K₀=1.26252±0.01896, #2213-2217
Chryseochelin C12:1
1+
395.25572
1+
801.37798
130.08712
215.09591
297.25817
569.27661
x10^5
+PASEF(815.39262), 35.0eV, 6.78-7.03min, 1/K₀=1.27900±0.01894, #2265-2346
Chryseochelin C13:1
1+
409.27024
1+
815.39198
1+
215.10293
100.07569
+PASEF(843.42323), 35.0eV, 7.34min, 1/K₀=1.35132±0.01887, #2448
Chryseochelin C15:1
1+
437.30091
1+
843.42594
100.07600
215.10284
322.29395
x10^4
+PASEF(871.45550), 35.0eV, 7.54-7.68min, 1/K₀=1.36116±0.01886, #2511-2558
Chryseochelin C17:1
1+
465.33246
1+
871.45295
1+
100.07558
1+
215.10279
m/z

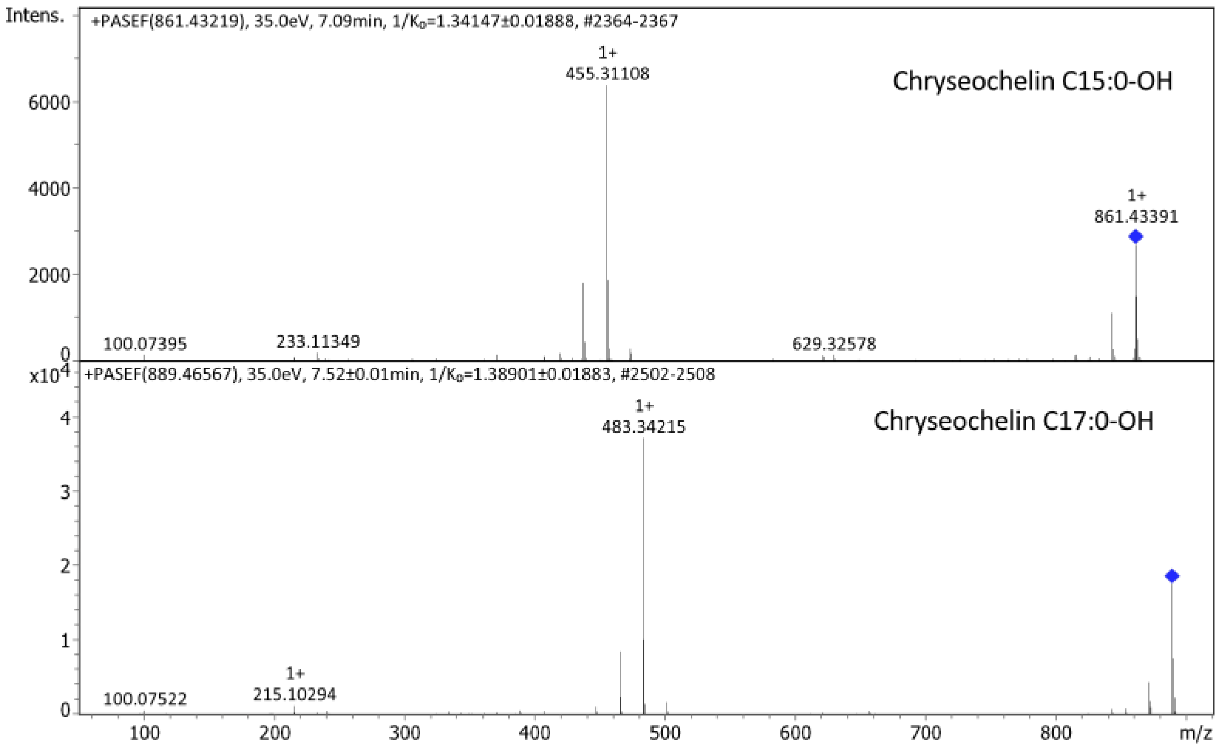

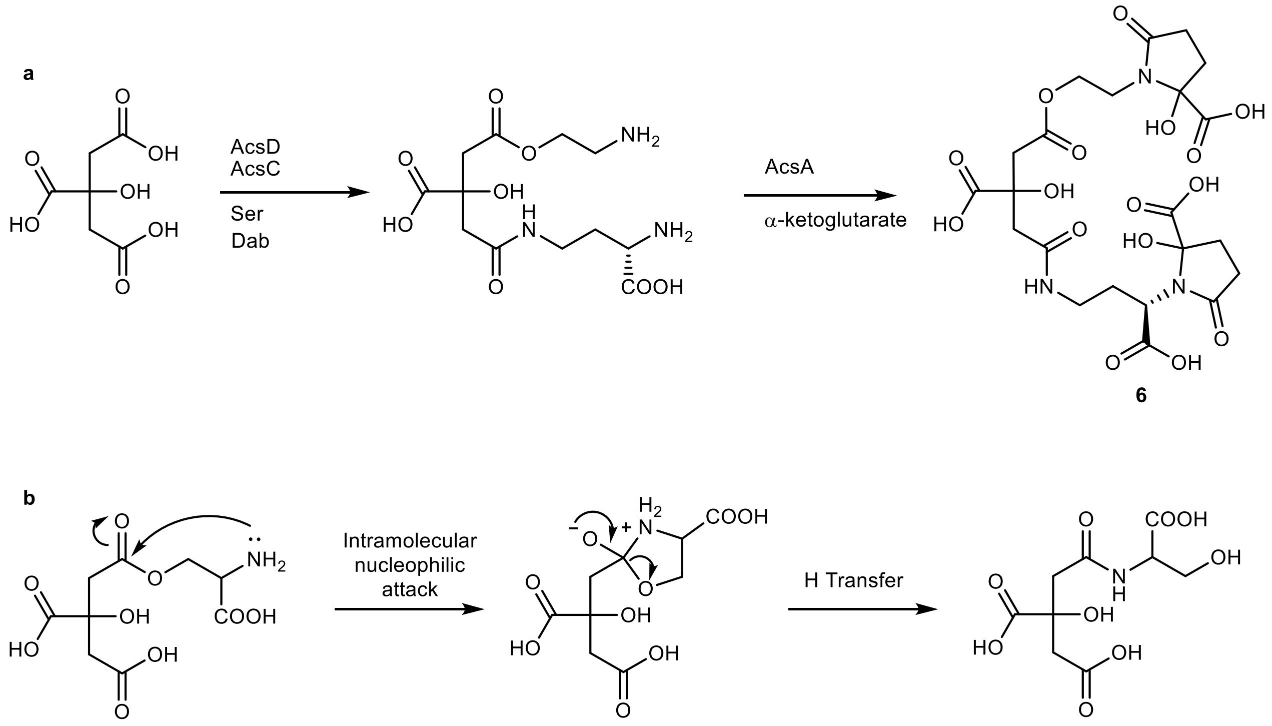

Figure S19. a) Major steps in the biosynthesis pathway of achromobactin (**6**). b) Re-arrangement reaction of the O-citryl-L-serine intermediate.

Chryseochelin A			Chryseochelin B			Chryseochelin $C_{15:0}$		
m/z	Intensity (a.u.)	Intensity (%)	*m/z*	Intensity (a.u.)	Intensity (%)	*m/z*	Intensity (a.u.)	Intensity (%)
82.06495	7926	9.6	82.06478	4752	2.8	99.00733	490	0.1
99.00787	2073	2.5	99.00748	5404	3.1	100.0755	19967	3.7
100.07560	20061	24.2	99.00764	5404	3.1	197.09227	6194	1.2
117.10203	10148	12.3	100.07571	25802	15	215.10342	49564	9.2
128.03414	7058	8.5	116.03404	8716	5.1	233.11344	5197	1
186.0759	5842	7.1	117.10239	11523	6.7	308.29633	5722	1.1
194.08159	5328	6.4	186.07609	24863	14.4	371.10986	5192	1
198.07609	6145	7.4	198.07608	7580	4.4	389.11861	10017	1.9
215.10249	82748	100	215.10322	172139	100	407.13065	8039	1.5
216.10592	8341	10.1	215.12078	8684	5	421.305	45817	8.5
227.10257	4233	5.1	216.10585	13773	8	422.30863	10023	1.9
233.11318	42104	50.9	233.11357	43983	25.6	437.30026	5624	1
234.11691	4919	5.9	234.11743	5939	3.5	438.30393	8027	1.5
307.09167	10227	12.4	257.11206	3726	2.2	439.31564	536743	100
325.10284	42129	50.9	307.09271	4779	2.8	439.34101	69009	12.9
326.1055	6384	7.7	325.10257	25900	15	440.31974	151138	28.2
335.08662	19991	24.2	335.08766	12797	7.4	440.34456	7109	1.3
343.11201	15639	18.9	343.11261	15672	9.1	441.32184	19197	3.6
343.12157	6110	7.4	344.11923	3794	2.2	457.3256	15530	2.9
353.09651	9004	10.9	347.10736	6153	3.6	613.33368	1330	0.2
361.12361	4505	5.4	353.0972	8978	5.2	621.22347	1743	0.3
371.10705	10100	12.2	361.12283	3813	2.2	747.4399	121	0.1
389.11921	6067	7.3	371.1089	19853	11.5	799.43029	10349	1.9
418.16007	4722	5.7	372.11169	3769	2.2	809.41897	7899	1.5
436.17066	6720	8.1	389.11884	68218	39.6	827.42691	60559	11.3
451.18115	9690	11.7	390.12143	10666	6.2	828.43104	28700	5.3
469.19269	14882	18	407.12982	16594	9.6	829.43514	7407	1.4
487.20064	6710	8.1	523.22306	1119	0.6	845.43904	171110	31.9
523.22621	137	0.2	549.18367	6536	3.8	846.44102	69976	13
531.17249	11295	13.6	557.20915	4272	2.5	847.44416	14648	2.7
549.18128	27001	32.6	567.19089	16916	9.8			
550.18397	7155	8.6	568.19502	3522	2			
557.20831	10672	12.9	575.21938	3990	2.3			
567.19314	48306	58.4	585.2033	16115	9.4			
568.19557	14507	17.5	586.20519	3858	2.2			
575.21687	8036	9.7	603.21443	36536	21.2			
585.20328	41352	50	604.2176	9374	5.4			
586.20519	11156	13.5	621.22435	163576	95			
603.21373	32561	39.3	621.25375	7654	4.4			
604.21379	11568	14	622.22742	48224	28			
621.22385	26024	31.4	623.23216	7384	4.3			
622.22759	8903	10.8						

Table S1. Peak list of MS/MS spectra illustrated in the main publication for chryseochelin A (intensity threshold above 5% unless essential fragment), chryseochelin B (intensity threshold above 2% unless essential fragment) and chryseochelin $C_{15:0}$ (intensity threshold above 1% unless essential fragment).

	Apo-Siderophore			Ferric Siderophore		
**Chryseochelin C Fatty Acid**	Neutral Sum Formula	*m/z* $[M+H]^+$	Retention Time [min]	Neutral Sum Formula	*m/z* $[M+H]^+$	Retention Time [min]
**C3:0**	C27H40N4O16	677.25121	4.72	C27H37N4O16Fe	730.1627	2.82
**C4:0**	C28H42N4O16	691.26686	5.18	C28H39N4O16Fe	744.1783	3.55
**C7:0**	C31H48N4O16	733.31381	5.84	C31H45N4O16Fe	786.2253	5.72
**C9:0**	C33H52N4O16	761.34511	6.24	C33H49N4O16Fe	814.2566	6.20
**C11:0**	C35H56N4O16	789.37641	6.61	C35H53N4O16Fe	842.2879	6.61
**C12:0**	C36H58N4O16	803.39206	6.81	C36H55N4O16Fe	856.3035	6.82
**C13:0**	C37H60N4O16	817.40771	7.02	C37H57N4O16Fe	870.3192	7.03
**C14:0**	C38H62N4O16	831.42336	7.22	C38H59N4O16Fe	884.3348	7.25
**C15:0**	C39H64N4O16	845.43901	7.44	C39H61N4O16Fe	898.3505	7.49
**C16:0**	C40H66N4O16	859.45466	7.72	C40H63N4O16Fe	912.3661	7.77
**C17:0**	C41H68N4O16	873.47031	7.85	C41H65N4O16Fe	926.3818	7.91
**C11:1**	C35H54N4O16	787.36076	6.44	C35H51N4O16Fe	840.2722	6.41
**C12:1**	C36H56N4O16	801.37640	6.61	C36H53N4O16Fe	854.2879	6.61
**C13:1**	C37H58N4O16	815.39206	6.81	C37H55N4O16Fe	868.3035	6.81
**C15:1**	C39H62N4O16	843.42336	7.16	C39H59N4O16Fe	896.3348	7.19
**C17:1**	C41H66N4O16	871.45466	7.56	C41H63N4O16Fe	924.3661	7.60
**C15:0-OH**	C39H64N4O17	861.43392	7.09	C39H61N4O17Fe	914.3454	7.09
**C17:0-OH**	C41H68N4O17	889.46522	7.53	C41H65N4O17Fe	942.3767	7.56

Table S2. Sum formulas, exact masses, and retention times for chryseochelin C derivatives in their apo and ferric state.
